# CRISPR-Cas9-generated *PTCHD1* 2489T>G stem cells recapitulate patient phenotype when undergoing neural induction

**DOI:** 10.1016/j.xhgg.2023.100257

**Published:** 2023-11-24

**Authors:** Kathryn O. Farley, Catherine A. Forbes, Nicole C. Shaw, Emma Kuzminski, Michelle Ward, Gareth Baynam, Timo Lassmann, Vanessa S. Fear

**Affiliations:** 1 Computational Biology, Precision Health, Telethon Kids Institute, Perth Children’s Hospital, Nedlands, WA 6009, Australia; 2 Translational Genetics, Precision Health, Telethon Kids Institute, Perth Children’s Hospital, Nedlands, WA 6009, Australia; 3 Centre for Child Health Research, University of Western Australia, Nedlands, WA 6009, Australia; 4 Western Australian Register of Developmental Anomalies, King Edward Memorial Hospital, Subiaco, WA 6008, Australia; 5 Undiagnosed Diseases Program, Genetic Services of WA, Subiaco WA 6008, Australia; 6 Rare Care Centre, Perth Children’s Hospital, Nedlands, WA 6009, Australia

**Keywords:** *PTCHD1*, rare disease, CRISPR-Cas9, homology-directed repair, disease modeling, synaptic dysfunction

## Abstract

An estimated 3.5%–5.9% of the global population live with rare diseases, and approximately 80% of these diseases have a genetic cause. Rare genetic diseases are difficult to diagnose, with some affected individuals experiencing diagnostic delays of 5–30 years. Next-generation sequencing has improved clinical diagnostic rates to 33%–48%. In a majority of cases, novel variants potentially causing the disease are discovered. These variants require functional validation in specialist laboratories, resulting in a diagnostic delay. In the interim, the finding is classified as a genetic variant of uncertain significance (VUS) and the affected individual remains undiagnosed. A VUS (*PTCHD1* c. 2489T>G) was identified in a child with autistic behavior, global developmental delay, and hypotonia. Loss of function mutations in *PTCHD1* are associated with autism spectrum disorder and intellectual disability; however, the molecular function of *PTCHD1* and its role in neurodevelopmental disease is unknown. Here, we apply CRISPR gene editing and induced pluripotent stem cell (iPSC) neural disease modeling to assess the variant. During differentiation from iPSCs to neural progenitors, we detect subtle but significant gene signatures in synaptic transmission and muscle contraction pathways. Our work supports the causal link between the genetic variant and the child’s phenotype, providing evidence for the variant to be considered a pathogenic variant according to the American College of Medical Genetics and Genomics guidelines. In addition, our study provides molecular data on the role of *PTCHD1* in the context of other neurodevelopmental disorders.

## Teaser

Deletions of *PTCHD1* have been associated with intellectual disability and autism. Here, Farley et al. report the functional consequences of a *PTCHD1* missense mutation leading to a neurodevelopmental disorder while further illuminating the mechanisms of *PTCHD1*-related neurodevelopmental disorders.

## Introduction

Mutations in the *PTCHD1* locus cause neurodevelopmental effects.^1^^,^^2^ However, little is known about the precise sequence of events leading to disease. *PTCHD1* is an X-linked gene mapping to Xp22.11^2^^,^^3^ and contains three coding exons. The 888-amino acid protein is a 12-pass transmembrane protein with a sterol-sensing domain.

The protein PTCHD1 shares a high degree of structural similarity with the Hedgehog (Hh) receptors Patched homolog 1 and 2 (PTCH1 and PTCH2). Three hedgehog genes have been described in vertebrates, the best characterized being Sonic hedgehog (*Shh*),^4^^,^^5^^,^^6^^–^^7^ which plays a vital role in patterning many systems, including neural tube formation and neuronal differentiation.^4^^,^^7^^,^^8^ PTCHD1, due to its structural similarity to PTCH1 and PTCH2, is thought to act as a Shh receptor.^9^^,^^10^^,^^11^ However, PTCHD1 also shares a PDZ domain seen in PTCHD4, suggesting a different mechanism for PTCHD1 dynamics, trafficking, and stability in synapses that relates to PDZ binding.^12^^,^^13^ The PDZ domain of PTCHD1 binds synapse-associated protein 102 and postsynaptic density protein 95.^12^^,^^13^^,^^14^ In addition, mouse models have found that SHH does not bind to PTCHD1,^12^^,^^13^ and PTCHD1 is unable to rescue PTCH1 deficiency.^13^ As such, PTCHD1 is not essential for SHH-dependent neuronal development and maintenance in mice.

In mouse models, *Ptchd1* deletion and truncating mutations in the thalamic reticular nucleus resulted in attention deficits and hyperactivity due to impaired small-conductance calcium-activated potassium channel conductance.^15^ A more severe phenotype is observed in mice with global loss of function mutations in *Ptchd1*, including learning impairment, cognitive defects, hyperactivity, hyperaggression, and motor defects, mimicking symptoms of attention-deficit/hyperactivity disorder (ADHD) and autism spectrum disorder (ASD), an umbrella term for a group of neurodevelopmental disabilities.^12^^,^^13^,^16^ Mouse models further support differing roles for *Ptchd1* in disease. Among these is a disruption of the synaptic excitatory/inhibitory (E/I) balance in *Ptchd1* knockout mice resulting from an impaired excitatory synaptic structure. In addition, *Ptchd1* deletion resulted in the upregulation of neuronal PAS domain protein 4 (*Npas4*) and early growth response 1 in mice.^13^ Overexpression of *Npas4* leads to increased inhibitory synapses, ultimately resulting in decreased hippocampal neurons.^17^^,^^18^^,^^19^

There are limited PTCHD1 studies in humans. Several studies indicate that PTCHD1 activity is modulated during neuronal activity,^16^^,^^20^ and variants in *PTCHD1* have been associated with a disruption in E/I balance.^2^^,^^12^^,^^13^^,^^15^^,^^16^ In humans, excitatory synapse function is impaired when the *PTCHD1* locus is disrupted.^16^ In addition, affected individuals with mutations in the region encompassing *PTCHD1* and the long noncoding RNA *PTCHD1-AS* (*PTCHD1-*antisense RNA [head to head]) demonstrate symptoms of ADHD, sleep disruption, hypotonia, aggression, ASD, and intellectual disability (ID).^1^^,^^2^^–^^3^^,^^16^^,^^21^^,^^22^^–^^23^ It is estimated that 1% of all people with ASD and ID have *PTCHD1* deletions and truncation mutations.^2^^,^^15^ Affected individuals present with a range of abnormalities involving communication, impaired social function, repetitive behaviors, and restricted interests.

**Figure 1:**
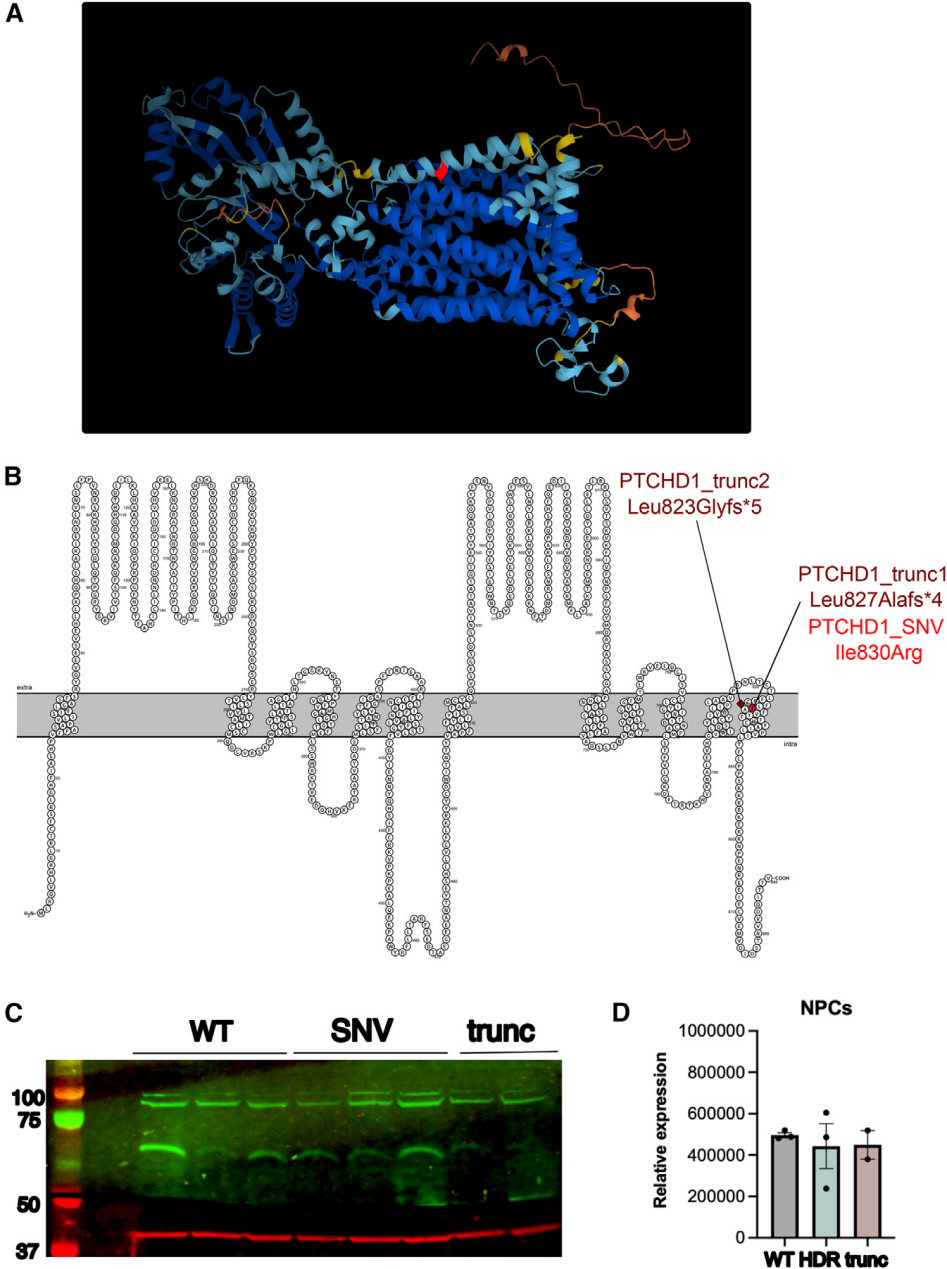
Structure and expression of PTCHD1 (A) AlphaFold model of PTCHD1 tertiary structure shows the 12 transmembrane domains present in PTCHD1, with the location of the patient VUS, Ile830Arg, in the 12th transmembrane domain highlighted in red. (B) Visualization of PTCHD1 primary structure was generated in Protter with transmembrane domains determined by deepTHMM. The variant of interest, Ile830Arg, is highlighted in red. Location of PTCHD1_trunc1 and 2 is noted, and predicted protein structure changes are visualized in Figure S2. (C) Expression of PTCHD1 and the loading control β-actin protein was analyzed in PTCHD1 NPC clones via western blot. (D) PTCHD1 expression was normalized to the β-actin loading control, with no significant difference observed between the PTCHD1_WT, SNV, and trunc groups (1-way ANOVA and Bonferroni’s correction for multiple testing).

A male child presented at 2 years of age with an undiagnosed rare disease and was found to have a variant of uncertain significance in the *PTCHD1* gene NM_173495 c.2489T>G (p.Ile830Arg). This nucleotide change is in the final exon of *PTCHD1*, and the predicted amino acid change is in the final transmembrane domain of PTCHD1. The affected individual’s phenotype was characterized using Human Phenotype Ontology terms and includes autistic behavior, global developmental delay, and muscular hypotonia (Table S1). Although prior investigations of *PTCHD1* mutations have been limited, this is in line with what we would expect to see given the phenotypes of other affected individuals with *PTCHD1* mutations (Table S2). Visualization of the structure of PTCHD1 predicted by AlphaFold^24^ is shown in Figure 1A. A 2D model of the protein structure with the patient variant of uncertain significance (VUS) highlighted is shown in Figure 1B. Prior studies in affected individuals with *PTCHD1* mutations have involved large-scale or whole-gene deletion, particularly within exon 1 and 2 of *PTCHD1*. Although *PTCHD1* missense mutations have previously been described, limited phenotypic information is available for these affected individuals.^22^ Interestingly, of the 13 missense mutations described by Halewa et al., Pro32Arg and Gly300Arg result in a change to arginine within a transmembrane domain, as does the mutation (Ile830Arg) of the affected individual we treated. However, of the three missense mutations described within the third exon of *PTCHD1,* none are within a transmembrane domain. As such, it is unknown what effect an SNV within the third *PTCHD1* exon—particularly one within the final transmembrane domain—would produce.

To investigate the role of this *PTCHD1* variant in patient phenotype, the patient variant and two truncating mutations were introduced into KOLF2-C1 (KOLF2) induced pluripotent stem cells (iPSCs) using CRISPR-Cas9 homology-directed repair (HDR).^25^^,^^26^^,^^27^^–^^28^ Post transfection, *PTCHD1* modifications were determined in polyclonal populations using amplicon sequencing.^29^^,^^30^^,^^31^^–^^32^ Polyclonal populations were then cloned to generate experimentally matched hemizygous clones containing the patient variant in *PTCHD1*, wild-type (WT) *PTCHD1*, or a truncated *PTCHD1* (herein referred to as *PTCHD1*_SNV, *PTCHD1*_WT, and *PTCHD1*_trunc; Figure S2).

The patient variant cells and healthy matched cells were differentiated into neural progenitor cells (NPCs) and their transcriptome profiled. We determined whether differentially expressed genes (DEGs) in edited versus healthy matched control cells are commensurate with the patient phenotype. In addition, *PTCHD1*_SNV was compared to *PTCHD1*_trunc to determine whether changes observed in *PTCHD1*_SNV cells align better with patient phenotype. Although these truncating mutations (p.(Leu827Alafs^∗^4) and p.(Leu823Glyfs^∗^5)) have not been previously classified as disease causing, they are predicted to truncate PTCHD1 by 58 and 61 amino acids, respectively. As such, they replicate the loss of the final transmembrane domain, including the C-terminal PDZ domain, and therefore form a good comparator to transcriptomic changes observed in the patient variant and WT clones. *PTCHD1*_SNV cells best recapitulated the affected individual’s phenotype, whereas a more severe phenotype was observed in *PTCHD1*_trunc cells. Altogether, we provide evidence to classify the patient variant as being a pathogenic variant according to American College of Medical Genetics and Genomics (ACMG) guidelines.^33^

## Material and methods

### Patient recruitment

Recruitment of the affected individual to this study was initiated by a genetic counselor based at Genetic Services Western Australia, followed by written informed consent. The study is in line with the Declaration of Helsinki and the National Health and Medical Research Council (NHMRC) National Statement on Ethical Conduct in Human Ethics Research and was approved by the Child and Adolescent Health Services, Human Research Ethics Committee (RGS0000000166). The patient genetic variant was determined with Massively Parallel Sequencing via Trusight One, and a genetic variant detected NM_173495(*PTCHD1*):c.[2489T>G]; 0 p(Ile830Arg)]; 0.

### Cell culture

KOLF2, a male iPSC line, was obtained from the Human Induced Pluripotent Stem Cell Initiative (HipSci). Cells were maintained in TeSR-E8 medium (StemCell Technologies, Australia) supplemented with 100 μg/mL Primocin (Invitrogen, Australia). Before transfection, cells were grown at 37°C in an atmosphere of 95% air and 5% CO_2_. Cells were passaged by washing in Dulbecco’s PBS and treated with gentle cell dissociation reagent (StemCell Technologies) for 2 min at 37°C. The dissociation reagent was carefully aspirated, and cells were resuspended in 1 mL TeSR-E8 and seeded at the desired density.

### CRISPR-Cas9 transfections

KOLF2 iPSCs grown to 30%–50% confluence were dissociated, seeded at a density of 1 × 10 ^5^ cells per well in 400 μL TeSR1 with 10 μM Y-27632 (StemCell Technologies) and placed at 32°C until the addition of the transfection mix. KOLF2 cell iPSC transfections were completed in 24-well plates precoated with Celladhere Vitronectin XF (StemCell Technologies).

Single guide RNA (sgRNA) was created by combining 1 μM CRISPR RNA (crRNA) AA (5′-CAAGCACCTGAACAGTGTAC-3′ PAM:AGG) or Ax (5′-ATCTGACCTGTACACTGTTC-3′ PAM:AGG) and 1 μM trans-activating crRNA in duplex buffer, incubated at 95°C for 5 min, and cooled to room temperature for 15 min. The ribonucleoprotein (RNP) complex was made by combining 63 nM sgRNA, OPTIMEM, Cas9 plus reagent, and 63 nM HiFi Cas9 endonuclease (IDT, Australia) and incubating for 5 min at room temperature. Antisense Alt-R HDR strand (5′-AAAGAAGGTGACAAATGCTCTTAAAAACAAGCACCTGAACAGTGTACATGTCAGATTTGAAGGCACAGCTGCAAGAGGA-3′) was added immediately post-RNP formation. RNP complexes were then transferred to a 1.5-mL Eppendorf tube containing OPTIMEM and STEM Lipofectamine and incubated for 10 min at room temperature.

Lipofectamine complexes were added dropwise to the wells to a final concentration of 21 nM of RNP complex, with 30 μM Alt-R HDR Enhancer (IDT). Stem cell medium was replaced daily, and the transfection plate moved to 37°C 48 h posttransfection. Cells were grown to confluence in 6-well plates and cryopreserved in knockout serum replacement media, or used for genomic DNA (gDNA) extraction (Invitrogen Purelink gDNA mini kit).

### Amplicon sequencing and cell cloning

Amplicon sequencing was completed on the MiniSeq (in-house; Illumina, US) sequencing system, as previously described.^30^^,^^31^ Briefly, a 247 bp target region was amplified via an initial PCR with *PTCHD1* amplicon primers *PTCHD1* AMPF1 (5′-AAAAATGCCCTGGAAGTG-3′) and *PTCHD1* AMPR1 (5′-ACTCAATTTCCTCCCGGTTCC-3′), linked to adaptor arms for PCR2 for the addition of barcodes.^32^ The MidOutput MiniSeq Kit (Illumina) was used for 150-bp paired-end, >10,000 reads, 300 cycles sequencing. Reads were analyzed with CRISPResso2^29^ for alignment with the *PTCHD1* WT or HDR amplicons.

*PTCHD1* transfected cells were single-cell cloned by limiting dilution. Once confluent in 24-well plates, cells were cryopreserved in knockout serum reagent with 10% DMSO or DNA lysates prepared for amplicon sequencing. For each cell sample, DNA lysate was prepared by suspension in 0.001 mM Tris pH 7, 100 μg Proteinase K (Applied Biosystems, Australia), and 50 μg/100 μL RNaseA (Invitrogen) and incubation at 56°C, 30 min, 96°C, 5 min. DNA lysates were screened with amplicon sequencing.

Three *PTCHD1* WT, three HDR, and two truncated experimentally matched clones were obtained. WT clones were called *PTCHD1*_WT_1, *PTCHD1*_WT_2, and *PTCHD1*_WT_3. *PTCHD1*_SNV_1, *PTCHD1*_SNV_2, and *PTCHD1*_SNV_3 contained the patient VUS. *PTCHD1*_trunc_1 and *PTCHD1*_trunc_2 were confirmed as independent clones predicted to truncate *PTCHD1* protein based on the mutations (NM_173495.2(*PTCHD1*_i001):p.(Leu827Alafs^∗^4) and NM_173495.2(*PTCHD1*_i001):p.(Leu823Glyfs^∗^5), respectively, generated.

### Clone screening

The top six off-target sites for crRNA AA or crRNA Ax were assessed in their relevant clones (Table S3). PCR reactions were performed with DreamTAQ HS Green master mix (Life Technologies), purified with Ampure XP beads, and Sanger sequencing performed (AGRF, Australia).

gDNA from each clone was screened using the StemCell Technologies hPSC Genetic Analysis Kit and qPCR analysis tool. In addition, cells from *PTCHD1*_SNV1 and *PTCHD1*_SNV2 clones were submitted to PathWest (Australia) for traditional cytogenetics analysis. Briefly, cells were grown in two independent 2 mL tissue culture tubes coated with Matrigel until 75% confluent and transferred to PathWest for conventional cytogenetics and G-banding. Five metaphases were analyzed per clone at a resolution of 400 bands per haploid set.

### Neural induction

iPSCs were differentiated into NPCs using the STEMdiff SMADi neural induction kit (StemCell Technologies), according to manufacturer’s instructions for monolayer culture. Briefly, for each clone, 1 × 10^6^ iPSCs were centrifuged and resuspended in neural induction medium (NIM) + SMADi + 10 μM Y-27632 and plated on fresh Matrigel-coated wells. Media was changed daily with NIM + SMADi and cells were passaged every sixth day, with cells being rinsed in DMEM/F-12, HEPES (Thermo Scientific, Australia) and dissociated with 1 mL of Accutase (StemCell Technologies) prewarmed to 37°C before resuspension in DMEM/F-12, HEPES, and centrifugation. After the third passage on day 18, cells were resuspended in neural progenitor medium (Figure S3).

At days 0 and 24 of differentiation, cells from each clone were photographed using an Eclipse TS2 camera (Nikon), 1 × 10^6^ cells were taken for protein extraction and western blot analysis, and 2 × 10^6^ cells were taken for RNA extraction by RNeasy kit (Qiagen, Australia). An additional 5 × 10^5^ cells were taken at days 0, 12, and 24 of differentiation and used for fluorescence-activated cell sorting analysis after FVS780 live/dead staining, fixation, and permeabilization (transcription factor staining buffer set, eBioscience, US), and antibody staining for stem markers OCT3/4-AF488 (BD Biosciences, US) and NanoG-BV421 (BioLegend, Australia), and neural markers PAX6-PE (BD Biosciences) and Nestin-AF647 (BioLegend). Samples were collected on an LSR Fortessa flow cytometer (BD Biosciences) and analyzed with FlowJo software (TreeStar, US). Gating strategy was total cells, with subsequent gating on live cells and then singles, before determination of percentage frequency protein marker expression.

### Protein extraction and western blot analysis

Proteins were collected using 1× Pierce immunoprecipitation lysis buffer with Pierce protease inhibitor (Thermo Scientific). Samples were quantified using the Millipore Direct Detect Spectrometer and diluted to equal concentrations. Samples were electrophoresed on a NuPAGE 4%–12% Bis-Tris gel (Invitrogen) and transferred to a 0.2-μm polyvinylidene fluoride membrane (BioRad). Membranes were blocked with LI-COR blocking buffer at 4°C overnight and stained with goat anti-Ptchd1 (1:500, NBP1-52108, Novus Biologicals) and β-actin (1:1,000, MA5-15739, Invitrogen), followed by infrared dye 680RD goat anti-mouse (1:5,000, LI-COR) or infrared dye 800 CW donkey anti-goat (1:5,000, LI-COR) and imaged using the Odyssey imaging system (LI-COR). Target protein signal was normalized to the β-actin signal for each sample using the housekeeping protein strategy. The lane normalization factor was determined by dividing the housekeeping protein signal for each lane by the housekeeping signal from the lane with the highest housekeeping protein signal. The normalized signal for each lane was then determined by dividing the target signal for each lane by the lane normalization factor.

### RNA sequencing (RNA-seq)

RNA integrity was determined on the Agilent 4200 Tapestation System with High Sensitivity RNA ScreenTape analysis. RNA-seq was performed according to the SureSelect Strand-Specific RNA library preparation protocol for Illumina Multiplexed Sequencing. Paired-end 100-bp libraries were sequenced to a depth of 30 million reads using Illumina’s NOVAseq 6000 platform (Genomics, Australia).

### Preprocessing, exploratory data analysis, and differential expression analysis

#### Data preprocessing

Raw sequencing reads were processed using the ENCODE/DCC pipeline (https://github.com/ENCODE-DCC/rna-seq-pipeline) in WDL (https://github.com/openwdl/wdl) via the Cromwell wrapper software Caper (https://github.com/ENCODE-DCC/caper). Within the pipeline, reads were aligned to GRCh39 and Kallisto^34^ abundance estimations used for gene quantification. Gene counts were read into R version 4.2.0 using the tximport^35^ package and gene expression tables created for downstream analysis with limma.^36^

#### Differential gene expression

Genes were filtered using the filterByExpr function,^37^ accounting for the number of samples in each group, and limma-trend models were fit to normalized log2 counts per million transformed data, calculated using the cpm function with a prior count of 3.^36^ Genes and transcripts with Benjamini-Hochberg corrected p values of less than 0.05 and absolute log fold changes of at least 0.5 were deemed to be differentially expressed. Principal-component analysis (PCA) plots were produced based on the top 500 variable genes.

#### Enrichment analysis

Functional gene set enrichment analysis (GSEA) was completed on all genes with the limma (limma:3.52.2); camera method via EGSEA (version 1.24.0)^38^ using moderated t-statistics, precision weights, and log fold changes from limma-voom. The GeneSetDB project Gene Ontology (GO), Pathway, Disease/Phenotype, and Human MSigDB c5 gene set collections were queried. In addition, clusterProfiler^39^ was used to compare functional profiles between genotypes, and disease associations were analyzed with DOSE.^40^

### Comparison to publicly available RNAseq data

RNA-seq data for WT NPCs were extracted from version 2.1 of ARCHS4 human gene level expression data using h5read.^41^^,^^42^ Genes were filtered, limma-trend models fit, and PCA plot generated, as described above.

Average expression was extracted from raw counts and log-transformed for both ARCHS4 and experimentally derived NPCs. Counts were visualized and the expression of key genes highlighted.

## Results

### Variant of uncertain significance in the *PTCHD1* gene

We introduced the patient VUS into iPSCs via CRISPR-Cas9 HDR using two crRNA strands and an antisense HDR template. Use of crRNA AA and crRNA Ax paired with the antisense HDR single-stranded DNA resulted in HDR frequencis of 8.6% and 6.0%, respectively, from which we derived single-cell clones. Three hemizygous *PTCHD1*_SNV clones were generated, alongside three experimentally matched *PTCHD1*_WT clones and two *PTCHD1*_trunc clones.

Next, iPSC clones were screened using Sanger sequencing across crRNA off-target sites and determined sequence integrity (Figure S4). Traditional karyotyping of *PTCHD1*_SNV1 and *PTCHD1*_SNV2 did not identify any chromosomal abnormalities in *PTCHD1*_SNV clones post transfection (Figure S5). In addition, all of the clones were assessed with karyotyping qPCR and were identical to the parental cell line (Figure S6).

Western blot of *PTCHD1*_WT, SNV, and trunc NPCs was then performed to determine changes in *PTCHD1* protein expression levels (Figures 1C and 1D). The expression of *PTCHD1* protein was not significantly higher in *PTCHD1*_WT cells compared to *PTCHD1*_SNV and *PTCHD1*_trunc derived NPCs (Figure 1D).

These data demonstrate the successful introduction of *PTCHD1*_SNV and *PTCHD1*_trunc into iPSCs via CRISPR-Cas9 HDR without producing off-target effects or inducing chromosomal changes. In addition, there were no significant changes in PTCHD1 protein expression at the NPC level.

### iPSC to NPC differentiation

**Figure 2:**
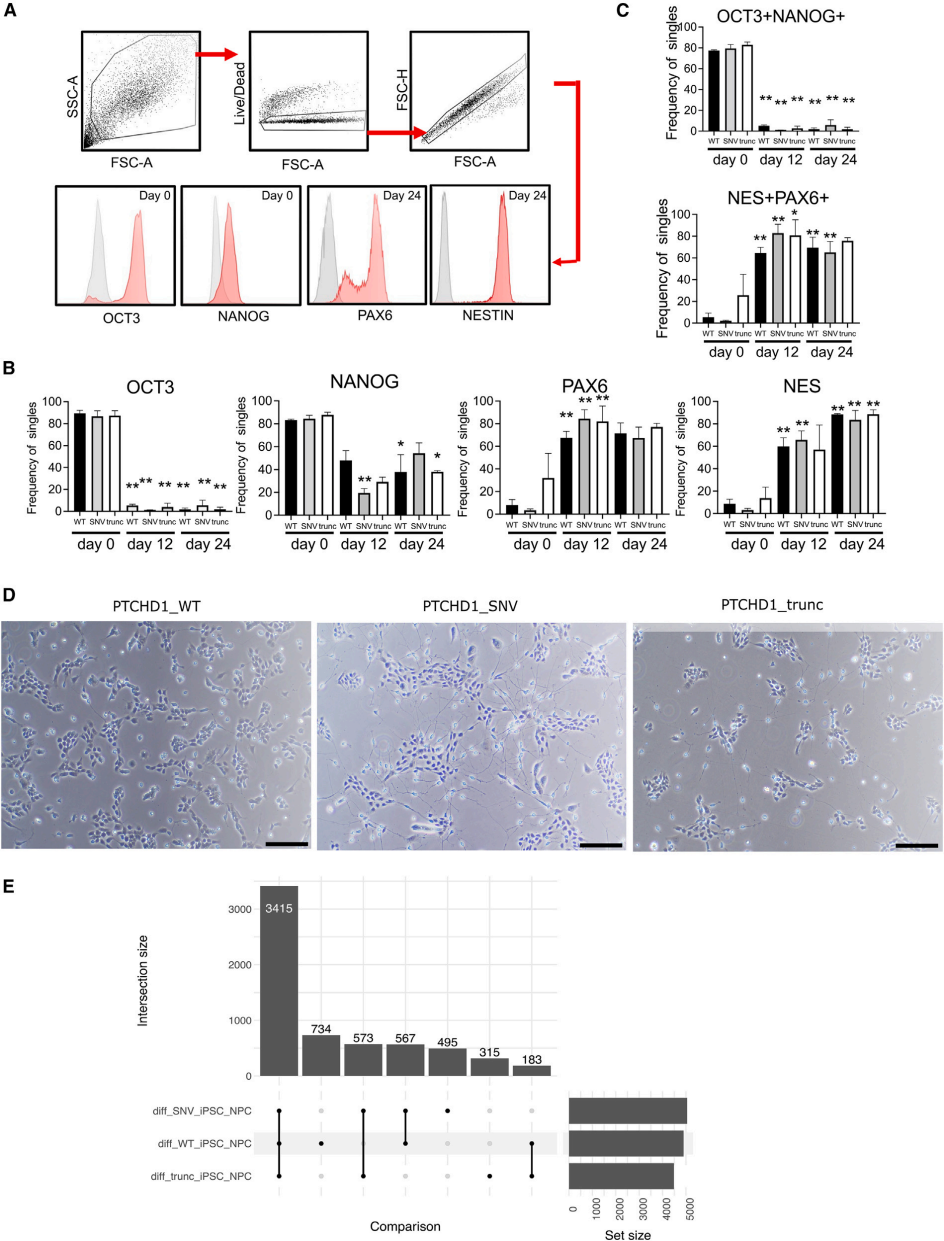
Neural differentiation (A) Cell-gating strategy as cells, live cells, single cells, and cell marker expression in histograms. (B) Bar graphs indicate frequency of live single cells positive for OCT3, NANOG, PAX6, and NESTIN (NES). (C) Bar graphs indicate frequency of live single cells that were OCT3^+^NANOG^+^ or PAX6^+^NESTIN^+^. ∗p < 0.05, ∗∗p < 0.01 compared to respective day 0 sample, Ordinary 1-way ANOVA with Sidak’s multiple comparison test. (D) Light microscopy images of PTCHD1 clones at day 24 of neural induction. Black bar, 100 μm. (E) Upset plot indicates number of DEGs for PTCHD1_WT, PTCHD1_SNV, and PTCHD1_trunc during differentiation of iPSCs into NPCs. A total of 3,415 DEGs were common to the 3 groups during differentiation.

*PTCHD1*_WT, *PTCHD1*_SNV, and *PTCHD1*_trunc cells underwent neural induction to generate NPCs (Figure S3). Flow cytometry analysis showed downregulation of the stem markers NanoG and OCT3/4 and upregulation of the neural markers Nestin and PAX6 in differentiation from iPSCs to NPCs, with similar values observed at the level of percentage frequency expression and mean fluorescence intensity in *PTCHD1*_WT, *PTCHD1*_SNV, and *PTCHD1*_trunc iPSCs and NPCs (Figures 2A–2C; Figure S7). In addition, cellular morphology confirmed differentiation into NPCs, with radial alignments and bipolar morphology identified in *PTCHD1*_WT, *PTCHD1*_SNV, and *PTCHD1*_trunc NPCs. Interestingly, an increase in neuronal processes was observed by eye in *PTCHD1*_SNV NPCs when compared to *PTCHD1*_WT and *PTCHD1*_trunc NPCs (Figure 2D).

Based on transcriptomics, PCA plot showed clear separation of iPSCs and NPCs (Figure S9). DEGs were found for all three sample groups during differentiation. *PTCHD1*_WT, *PTCHD1*_SNV, and *PTCHD1*_trunc had 4,899, 5,049, and 4,486 significant DEGs, respectively, with 3,415 genes common to all 3 (Figure 2E; and Tables S4–S7). In keeping with protein expression levels, the gene expression of *PTCHD1* was not significantly different at the iPSC or NPC stage when comparing *PTCHD1*_WT, *PTCHD1*_SNV, and *PTCHD1*_trunc cells (Figure S10).

Notably, there was a significant decrease in PTCHD1 during differentiation for each genotype. Furthermore, there was no statistically significant difference in expression of the NPC markers Nestin, PAX6, and SOX1 and markers for potentially contaminating neural crest cells (TUBB3 and NGFR; Figure S10).

To ensure our NPCs were comparable to WT NPCs, we integrated our transcriptome with publicly available data. PCA showed that *PTCHD1*_WT, *PTCHD1*_ SNV, and *PTCHD1*_trunc NPCs grouped with WT NPCs in the ARCHS4 database (Figure S11).^42^ When examining expression profiles between the two groups, expression of TUBB3, SOX1, PAX6, and Nestin was comparable (Figure S12).

GSEA showed that multiple gene sets were commonly enriched in *PTCHD1*_WT, *PTCHD1*_SNV, and *PTCHD1*_trunc during neural differentiation, with gene sets including “GO positive regulation of neural precursor cell proliferation” (adjusted p = 0.03), “GO Neuron fate specification” (adjusted p = 0.03), and “GO neural precursor cell proliferation” (adjusted p = 0.04) being upregulated (Figure S13). Overall, of the top 20 upregulated gene sets, 18 were related to neural differentiation, patterning, and development (Table S8).

Together, these data indicated that *PTCHD1*_WT, *PTCHD1*_SNV, and *PTCHD1*_trunc cells successfully differentiated into NPCs closely resembling WT NPCs in their global transcriptome.

### Gene signatures in cells containing the patient variant match the patient phenotype

We compared *PTCHD1*_SNV, *PTCHD1*_WT, and *PTCHD1*_trunc cells to discover DEGs. No DEGs reached significance in the comparison of WT, SNV and trunc cells at both the iPSC and NPC stage. However, significant DEGs were observed for all three *PTCHD1* genotypes during differentiation (Figures 2E and S14).

**Figure 3:**
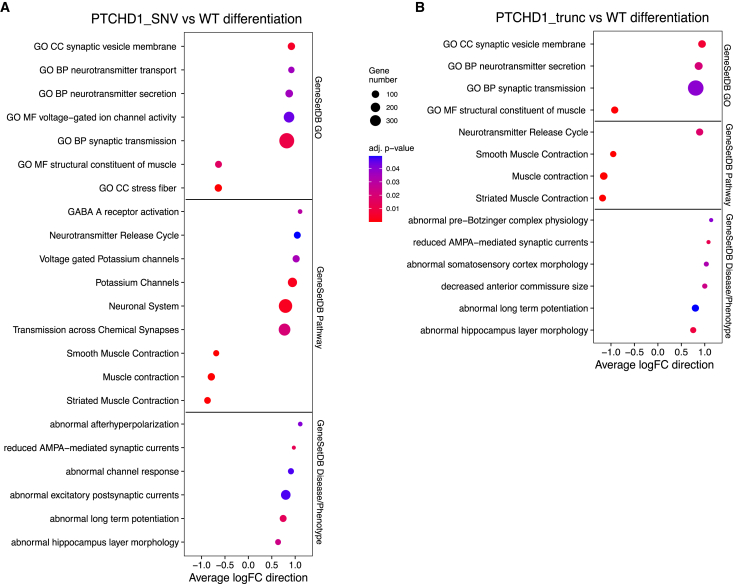
Difference in expression during differentiation for PTCHD1_WT, PTCHD1_SNV and PTCHD1_trunc cells (A) Bubble plots of GSEA results for the comparison of PTCHD1_SNV to PTCHD1_WT cells during differentiation show increased downregulation of muscle contraction and upregulation of synaptic dysfunction–related gene sets such as abnormal afterhyperpolarization and reduced AMPA-mediated synaptic currents in PTCHD1_SNV cells. (B) Bubble plots of GSEA results for the comparison of PTCHD1_trunc to PTCHD1_WT cells during differentiation. Analysis indicated enrichment of similar gene sets to what is observed in the comparison of PTCHD1_SNV to PTCHD1_WT during differentiation, albeit more severe gene sets such as abnormal pre-Bötzinger complex physiology were upregulated in PTCHD1_trunc, and these are not relevant to patient phenotype.

To test whether whole biological pathways or groups of disease genes are dysregulated, we performed GSEA. To test whether mutations in *PTCHD1* affect gene expression during differentiation into neural progenitors, we compared the changes observed in WT samples to the changes observed in edited cells. Enriched gene sets included upregulation of voltage-gated ion channel activity and neurotransmitter secretion and transport. Similarly, voltage-gated potassium channels and transmission across chemical synapses were upregulated in the GeneSetDB Pathway gene sets. Concurrently, striated muscle contraction, muscle contraction, and smooth muscle contraction were downregulated. Gene sets relating to signal transmission, including abnormal long-term potentiation (LTP), were upregulated (Figure 3A; Tables S9–S11).

In addition, we compared changes observed in WT samples to the changes observed in cells containing truncating mutations. Similar results were observed, with upregulation of neurotransmitter-related gene sets. In addition, muscle contraction, striated muscle contraction, and smooth muscle contraction were downregulated and the top-ranked gene sets. Further disease/phenotype gene sets were present, including abnormal pre-Bötzinger complex physiology, abnormal somatosensory cortex morphology, and decreased anterior commissure size being upregulated (Figure 3B).

Gene sets associated with the downregulation of muscle contraction were enriched in both *PTCHD1*_SNV and *PTCHD1*_trunc cells during differentiation. In addition, synapse-associated gene sets, including synaptic transmission, abnormal LTP, and reduced AMPA-mediated synaptic currents were upregulated. Other terms, such as abnormal pre-Bötzinger complex—critical for respiratory rhythm—and decreased anterior commissure size—a structure contributing to memory, emotion, speech, and hearing^43^—were found exclusively in *PTCHD1_*trunc cells. Interestingly, the difference between *PTCHD1*_WT and *PTCHD1*_SNV cells during differentiation showed a large number of terms related to synapse and synaptic membrane-related function (Figure S15). In addition, the most significant disease ontology term was brain disease (adjusted p = 4.66E−5), and further terms included ASD (adjusted p = 0.0052) and autistic disorder (adjusted p = 0.0052) (Figure S16; Table S12). Interestingly, in *PTCHD1*_trunc cells, there were 229 enriched disease ontology terms, with developmental disorder of mental health (adjusted p = 3.82E−5) being the most significant patient-relevant term and ranked 12th (Table S13).

These data indicated that when compared to *PTCHD1_*WT cells, *PTCHD1*_SNV cells undergoing differentiation best represented patient phenotype, with a similar, more severe phenotype being identified in the comparison of *PTCHD1_*trunc to *PTCHD1_*WT cells during differentiation.

## Discussion

Rare disease diagnosis is a difficult and lengthy process for affected individuals and their families. Here, we examined a *PTCHD1* VUS to determine its relevance to the phenotype of the affected individual we treated. We introduced the patient variant into iPSCs via CRISPR-Cas9 HDR, differentiated clonal genetic variant cells to NPCs, and identified changes in gene expression consistent with the affected individual’s phenotype.

Methods for editing cells using CRISPR-Cas9 now achieve high HDR rates, allowing us to utilize these molecular tools to study patient mutations.^44^^,^^45^^,^^30^^,^^31^^,^^46^^,^^47^^,^^48^^,^^49^ *PTCHD1*_SNV and *PTCHD1*_trunc were both introduced into stem cells without affecting karyotype or pluripotency (Figures 2A–2C, S5, and S6), enabling differentiation into the cell type of interest.

Stem cells successfully differentiated into NPCs regardless of the presence or absence of *PTCHD1* mutations. Morphologically, all of the cells presented similarly, although an increase in neuronal processes was visible in *PTCHD1*_SNV NPCs. It is estimated that 80% of ASD high-risk genes regulate processes involved in neurite growth.^50^ Although it is not the direct focus of this study, it is conceivable that *PTCHD1* has an as yet unknown role in processes relating to neurite growth.

Several lines of evidence suggest that the patient VUS is indeed responsible for disease. Upon differentiation, *PTCHD1*_SNV cells exhibited distinct changes compared to *PTCHD1*_WT cells. Many of these distinct changes were shared with *PTCHD1_*trunc cells, including upregulation of gene sets relating to synaptic transmission. The way in which the brain regulates synaptic transmission is vital to learning, memory, motor control, and sensory processing, and this relates to the balance of E/I transmissions. The ability of neurons to adjust the balance of E/I transmissions and the strength of these transmissions is known as synaptic plasticity.

Disruption of the E/I balance has a role in ASD,^51^^,^^52^^–^^53^ and this is seen in affected individuals with *PTCHD1* mutations.^2^^,^^12^^,^^13^^,^^15^^,^^16^ This disruption in E/I balance is primarily attributed to abnormal GABAergic and glutamatergic neurotransmission.^54^ A type of glutamate receptors known as AMPA receptors are responsible for the majority of fast excitatory synaptic transmission in the CNS.^55^ As such, reduction of AMPA-mediated synaptic currents will affect excitatory postsynaptic currents and contribute to a disruption in E/I balance.^56^^,^^57^^,^^58^^–^^59^ The gene set “reduced AMPA-mediated synaptic currents” was upregulated in *PTCHD1*_SNV and *PTCHD1_*trunc cells during differentiation compared to *PTCHD1*_WT cells. Concurrently, the gene set “GABA A receptor activation” was upregulated in *PTCHD1*_SNV cells during differentiation. Because GABA is the chief inhibitory neurotransmitter in the brain, this would lead to further disruption of the E/I balance in *PTCHD1*_SNV cells. This is consistent with prior *Ptchd1* mouse models^12^^,^^13^ in which the E/I balance was disrupted, leading to disease.

In addition, when comparing the changes observed in *PTCHD1*_SNV and *PTCHD1_*trunc cells to those seen in *PTCHD1*_WT cells, the gene set “abnormal long-term potentiation” is upregulated. LTP and long-term depression (LTD) are both involved in synaptic plasticity. Synaptic plasticity is believed to play a role in learning and memory.^60^ LTP results in the strengthening of a synapse, increasing signal transmission, whereas LTD reduces synaptic strength, thereby decreasing signal transmission. Abnormal LTP can cause hyper- and hypoplasticity, which have both been observed in people with ASD.^61^^,^^62^^,^^63^^–^^64^

Abnormal hippocampal layer morphology was upregulated when comparing *PTCHD1_*SNV and *PTCHD1_*trunc cells to *PTCHD1_*WT during differentiation. In *PTCHD1* knockout mouse models, structural and functional alterations were found in excitatory synapses of the hippocampus and hippocampal activity was impaired.^13^ Interestingly, the upregulation of potassium channels and abnormal afterhyperpolarization was a gene signature unique to the comparison of *PTCHD1*_SNV to *PTCHD1*_WT during differentiation. Afterhyperpolarization is the phase of a neuron’s action potential in which the cell’s membrane potential is lower than the normal resting potential and is governed by ion channels. Ion channel defects, or channelopathies, have been implicated in several neuropsychiatric disorders, including ID and ASD.^65^^,^^66^ Furthermore, gain- and loss-of-function mutations can cause potassium channelopathies, and several potassium channels have functions involving muscle contraction and controlling smooth muscle tone.^66^

Upregulation of the gene sets above, implicating the dysregulation of genes associated with synaptic transmission, is also a possible explanation for the dysregulation of genes associated with muscle contraction observed as being downregulated in *PTCHD1*_SNV and *PTCHD1*_trunc cells. Furthermore, this downregulation of muscle contraction–related gene sets represents another gene signature relevant to the patient phenotype, which includes central and muscular hypotonia. Muscle tone, or the amount of resistance to passive movement of a muscle, is related to the ability of a muscle to contract. If a muscle is unable to properly contract, then this can lead to hypotonia. Hypotonia is defined as a state of low muscle tone resulting in floppiness, with a key feature of central hypotonia being abnormal brain function. Muscular hypotonia typically presents with weakness, joint contractures, and decreased tendon reflexes.^67^ These data indicate that PTCHD1 mutations can modulate genes in these pathways.

Neural progenitor cells are immature neural cells incapable of the activities of fully functional neurons. However, NPCs have limited electrical activity and express voltage-gated ion channels. It is therefore possible that perturbations caused by the VUS will manifest more strongly in later stages of differentiation—for example, in glutamatergic or GABAergic neurons. Studying the effect of the variant in mature neurons could be a valuable direction for future studies.

In addition, it is possible that mutant PTCHD1 proteins traffic differently within cells and neurons. However, there is currently a lack of effective PTCHD1 antibodies available for use in protein localization studies such as immunohistochemistry.

Although the combination of differences in cellular morphology and the presence of synaptic-related terms could be attributed to a larger proportion of mature neurons being present in *PTCHD1*_SNV cultures compared to *PTCHD1*_WT cultures, given that there were no significant differences in the expression of NPC and neural crest cell markers between genotypes, this is unlikely.

A limited number of affected individuals with *PTCHD1* SNVs have been reported in the literature, and their phenotypic information is scarce.^2^^,^^22^ Additional studies in affected individuals with *PTCHD1* mutations have primarily described affected individuals with whole-exon or whole-gene deletions.^1^^,^^2^^–^^3^^,^^16^^,^^21^^,^^23^,^68^ These affected individuals share phenotypic similarities with those of the *PTCHD1*_SNV affected individual described here and outlined in Table S1, including hypotonia, ASD features, and global developmental delay (Table S2). In addition, facial dysmorphisms are common in affected individuals with *PTCHD1* mutations. Although these dysmorphisms are variably expressed, a long philtrum was observed in the proband and has been observed in several other affected individuals.^1^^,^^68^ This is further support for *PTCHD1*_SNV being disease causative in the child we treated.

Overall, the gene expression changes observed during the differentiation of cells containing the affected individual’s variants aligned better with their phenotype compared to changes observed in WT and the truncation genotype. Paired with the patient-relevant disease ontology terms enriched in *PTCHD1_*SNV cells but not *PTCHD1*_trunc cells, this supports the patient variant as being disease causative. ACMG criteria require several lines of evidence for a variant to be classified as pathogenic.^33^ The functional validation completed here provides PS3 evidence of pathogenicity. The hemizygous variant has not been inherited from the mother, as confirmed by whole-exome sequencing. As such, the variant is an assumed *de novo* variant, which is absent from controls. This fulfills the requirements for two moderate criteria (PM6 and PM2). Finally, the amino acid change (Ile830Arg) affects a moderately conserved amino acid, and multiple *in silico* algorithms support pathogenicity (Table S14). Collectively, this is evidence of the variant being pathogenic.

A possible limitation of our study is the fact that we used KOLF2-C1 cells, known to contain a 19-bp deletion in the ARID2 gene.^48^ However, we internally control for this because all of the samples in our work have the same genetic background. Therefore, observed changes cannot be attributed to the ARID2 mutation. Furthermore, we did not observe any major changes in the expression of ARID2 when comparing to WT NPCs from the ARCHS4 database (Figure S11) or within our data (Figure S9).

### Conclusions

We characterized the functional consequences of a *PTCHD1* variant of uncertain significance during early neuronal development. *PTCHD1_*SNV was implicated in widespread synaptic dysfunction when differentiating iPSCs into neural progenitors. Comparison to healthy controls and further lines of molecular evidence support the role of the variant in widespread synaptic dysfunction aligning well with the observed disease of the affected individual in whom the variant was discovered. Our results specifically point to excitatory synapse disruption and an impaired E/I balance as a consequence of *PTCHD1* mutations. Collectively, the results advance the understanding of the role of *PTCHD1* mutations in disease and provide evidence to classify this VUS as being pathogenic.

## Data and code availability

Raw FASTQ files and processed count data are available at the GEO repository under accession number GSE227711. ptchd1_paper_analysis.pdf contains all of the code to reproduce the analysis and figures in this paper.

## Supplemental information

Data S1. Tables S2 and S4–S13

Document S2. Article plus supplemental information
