# Supplementary material for: CRISPR-Cas9-generated PTCHD1 2489T>G stem cells recapitulate patient phenotype when undergoing neural induction
Source: HGG Adv. 2023 Nov 24;5(1):100257. doi: 10.1016/j.xhgg.2023.100257 (PMC10787298; doi:10.1016/j.xhgg.2023.100257)
Supplement: Document S1. Figures S1–S17 and Tables S1, S3, and S14 [file mmc1.pdf]

HGGA, Volume *5*

Supplemental information

CRISPR-Cas9-generated *PTCHD1* 2489T>G stem cells recapitulate patient phenotype when undergoing neural induction

Kathryn O. Farley, Catherine A. Forbes, Nicole C. Shaw, Emma Kuzminski, Michelle Ward, Gareth Baynam, Timo Lassmann, and Vanessa S. Fear

Figure S1

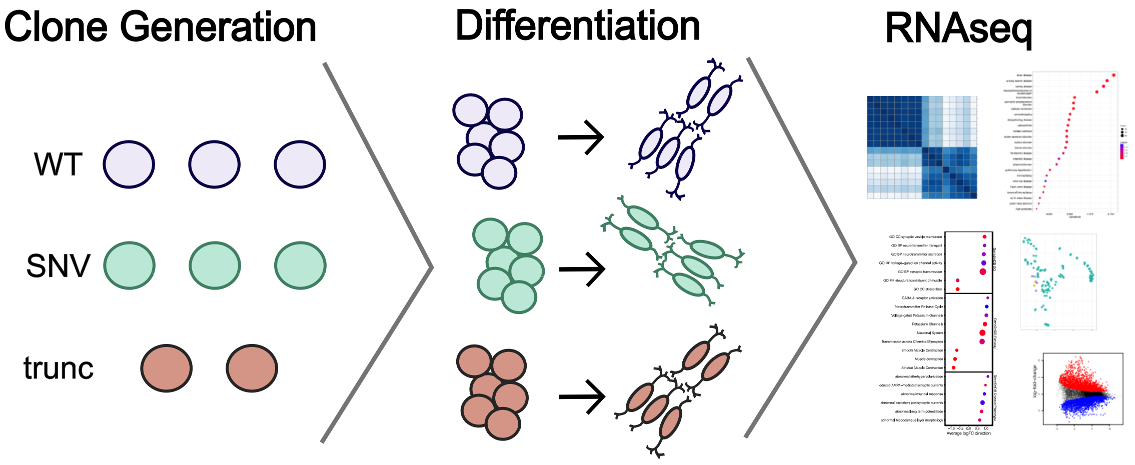

Experimental design. Three patient variant (SNV) iPSC clones were obtained using CRISPR/Cas9 gene editing and single cell cloning. Three experimentally matched wild-type clones, and two clones with truncating *PTCHD1* mutations were obtained. Obtained clones were differentiated to NPCs and RNAseq analysis completed.

Figure S2

a

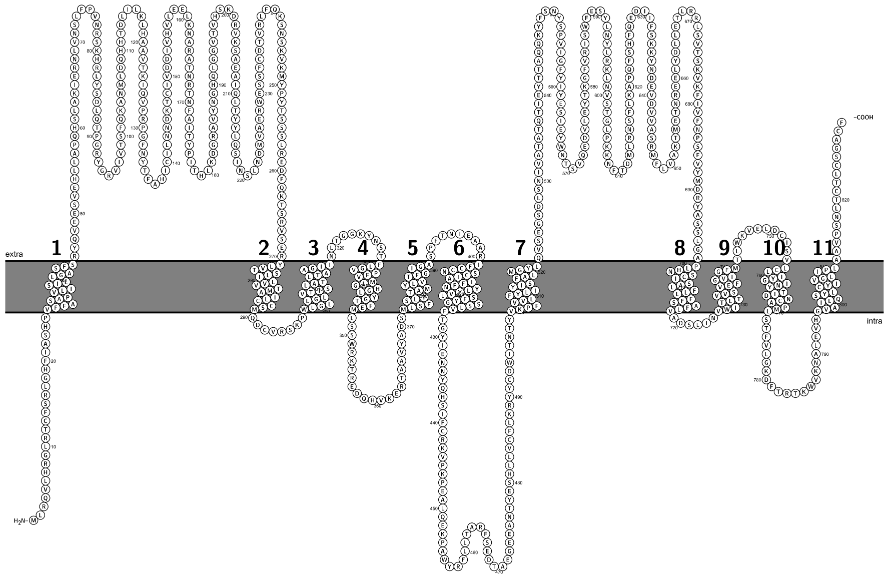

b

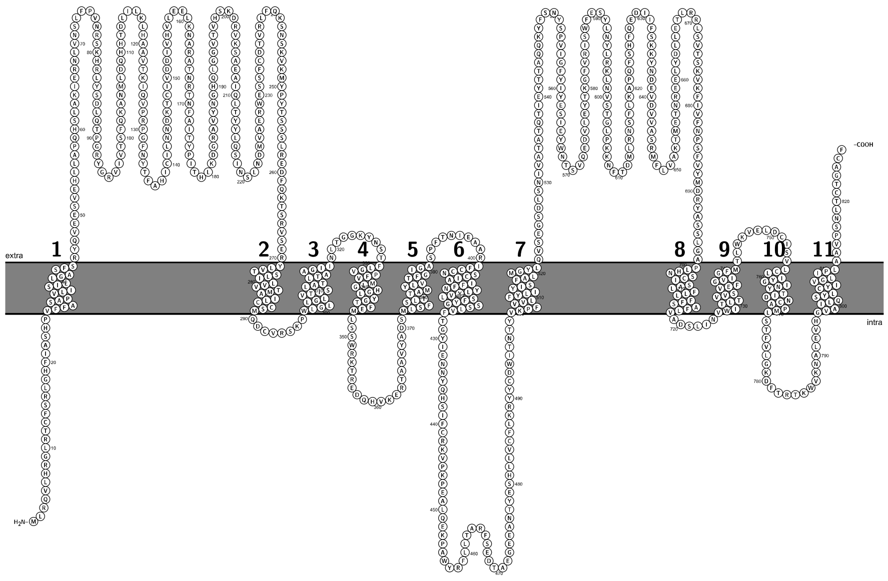

2D representations of the PTCHD1_trunc clone primary protein structure as predicted by deepTHMM and visualised in Protter. Both clones were predicted to produce a premature stop codon, disrupting the final transmembrane domain.

a *PTCHD1*_trunc 1 NM_173495.2(*PTCHD1*_i001):p.(Leu827Alafs*4)

b *PTCHD1*_trunc 2 NM_173495.2(*PTCHD1*_i001):p.(Leu823Glyfs*5)

Figure S3

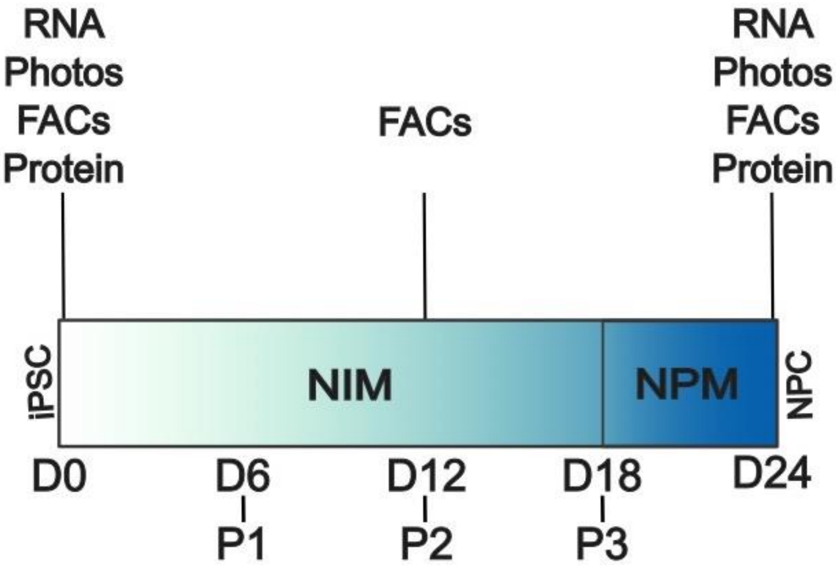

Generation of neural progenitor cells from iPSC clones. The three independent PTCHD1_SNV, and three experimentally matched PTCHD1_WT clones were differentiated to NPCs, with differentiation completed once for each pair. Additionally, PTCHD1_trunc1 and PTCHD1_trunc2 were experimentally matched to differentiation batches 1 and 2 respectively.

Figure S4

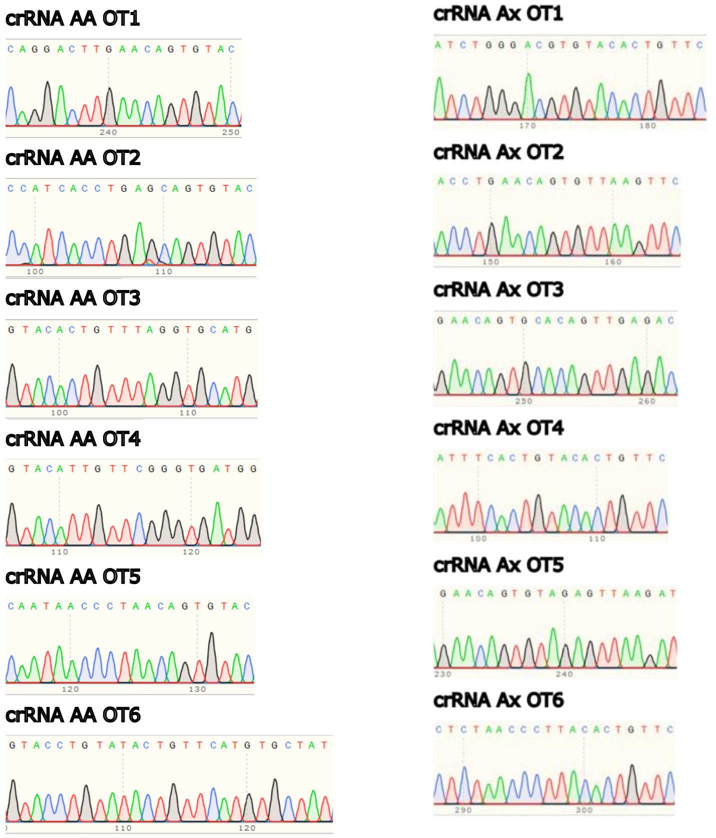

Off-target PCR chromatograms for crRNA AA and crRNA Ax. No off-target effects were found. Primer/target information can be viewed in supplementary table 3.

Figure S5

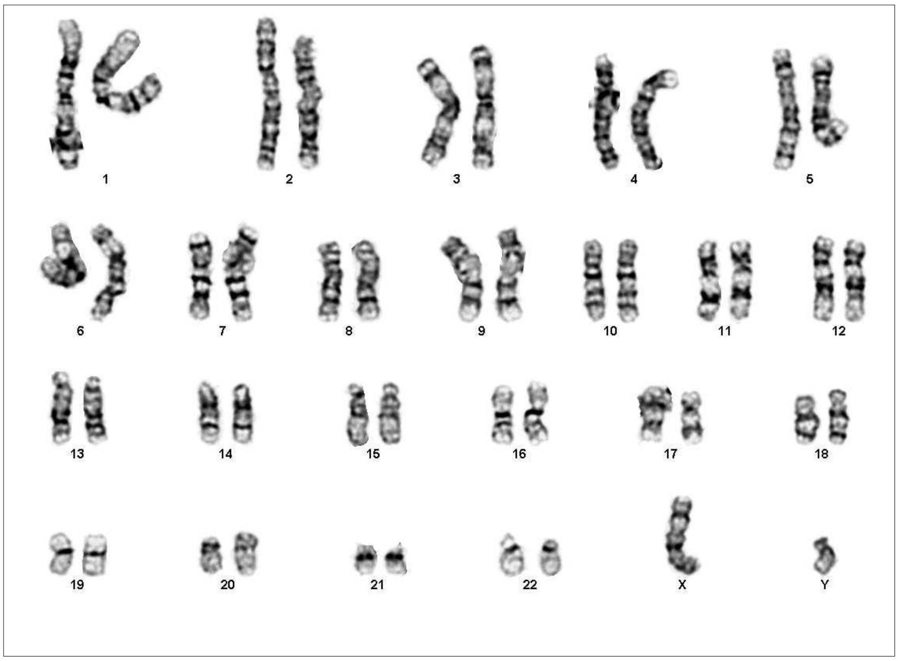

Karyotyping of KOLF2 cells containing *PTCHD1*_SNV determined a 46, XY karyotype. Cells were karyotyped in the Cytogenetics section, Diagnostic Genomics, PathWest.

Supplementary Figure 6.

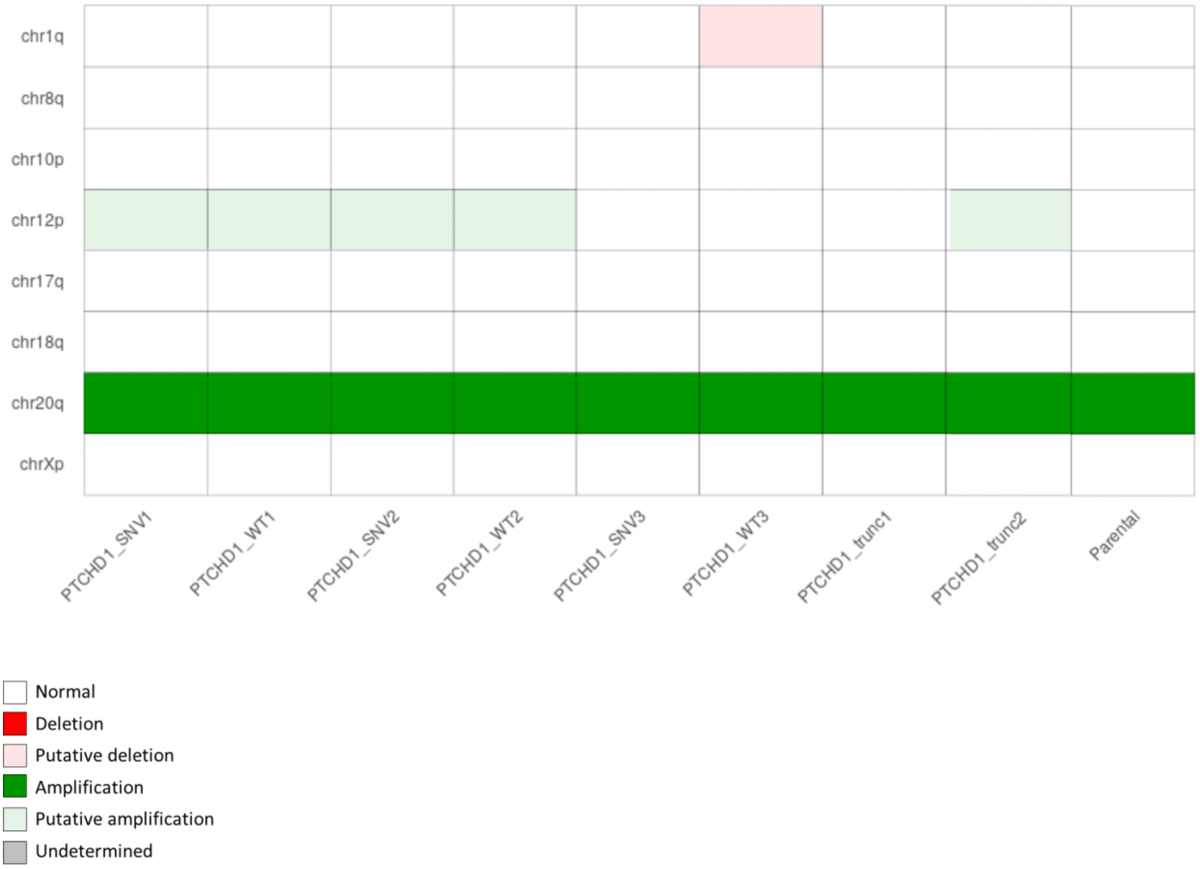

Karyotyping qPCR showed consistent karyotype amongst clones, including the parental KOLF-2 cell line.

Figure S7

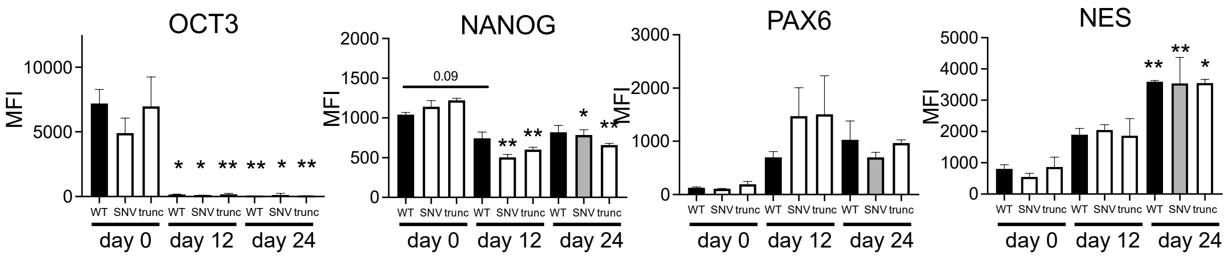

Neural differentiation, Geometric mean fluorescence intensity MFI, at indicated timepoints. *p<0.05, **p<0.01 compared to respective day 0 sample, Ordinary one-way ANOVA with Sidaks Multiple comparison test.

Figure S8

a

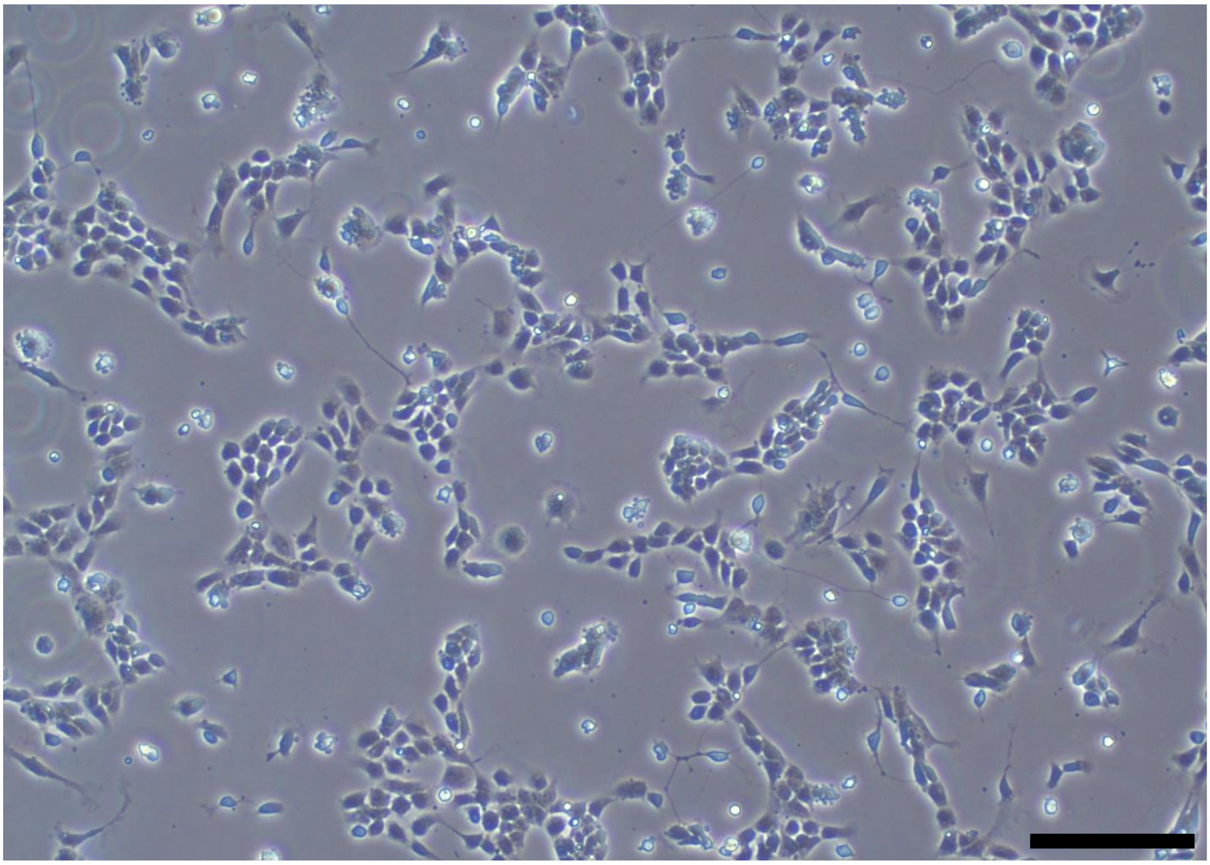

b

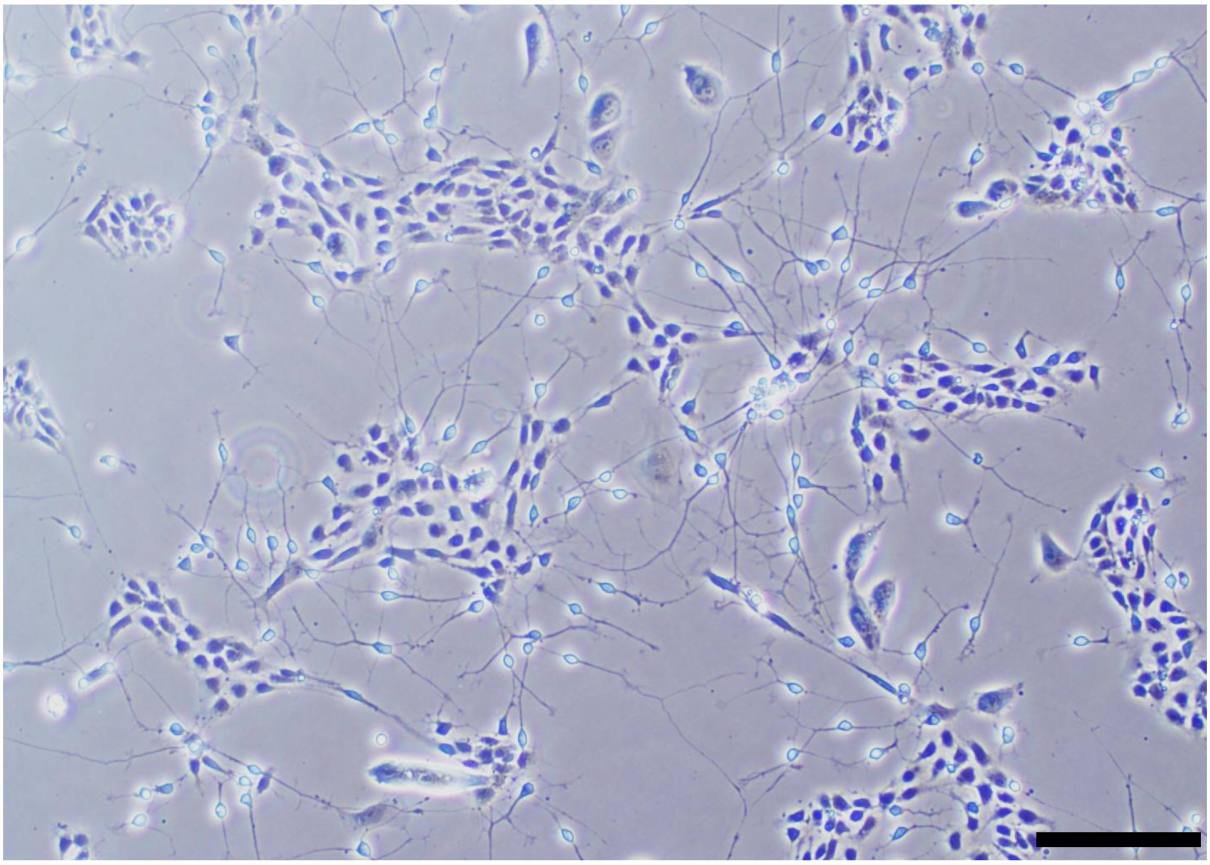

c

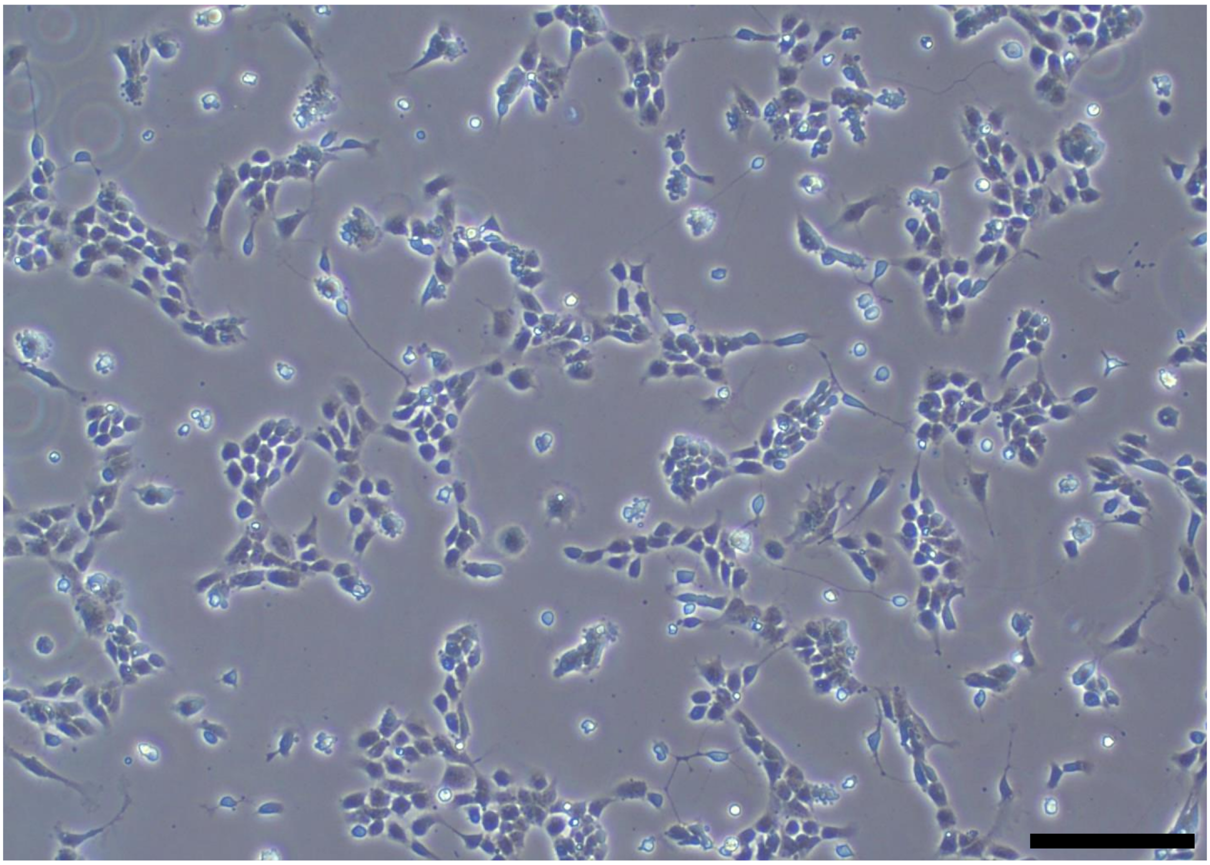

Light microscopy images of PTCHD1 clones at day 24 of neural induction. Black bar indicates 100 microns. **a** *PTCHD1*_WT **b** *PTCHD1*_SNV **c** *PTCHD1*_trunc

Figure S9

a.

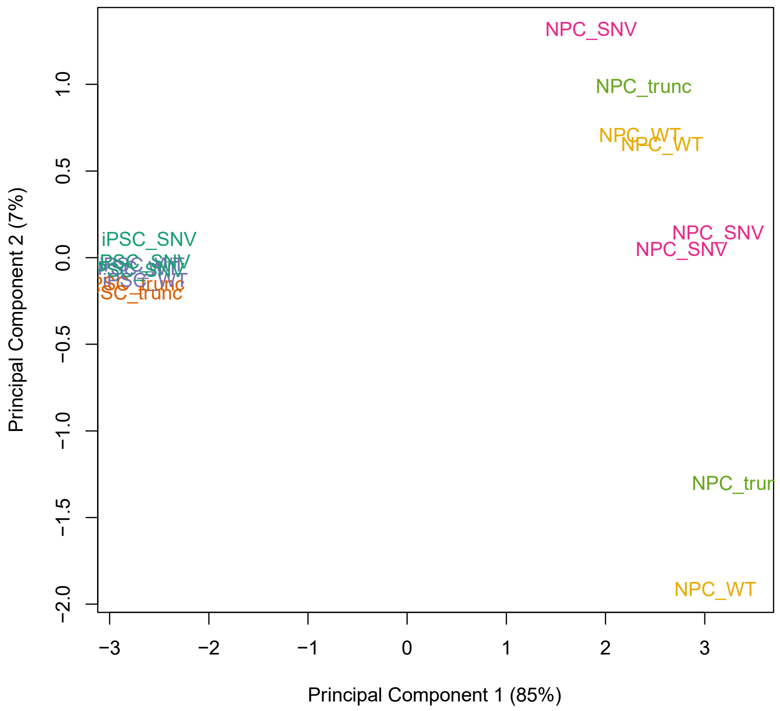
NPC_SNV
NPC_trunc
NPC_WT
NPC_SNV
NPC_SNV
iPSC_SNV
NPC_WT
1.0
0.5
0.0
−0.5
−1.0
−1.5
−2.0
−3
−2
−1
0
1
2
3
Principal Component 2 (7%)
Principal Component 1 (85%)

b.

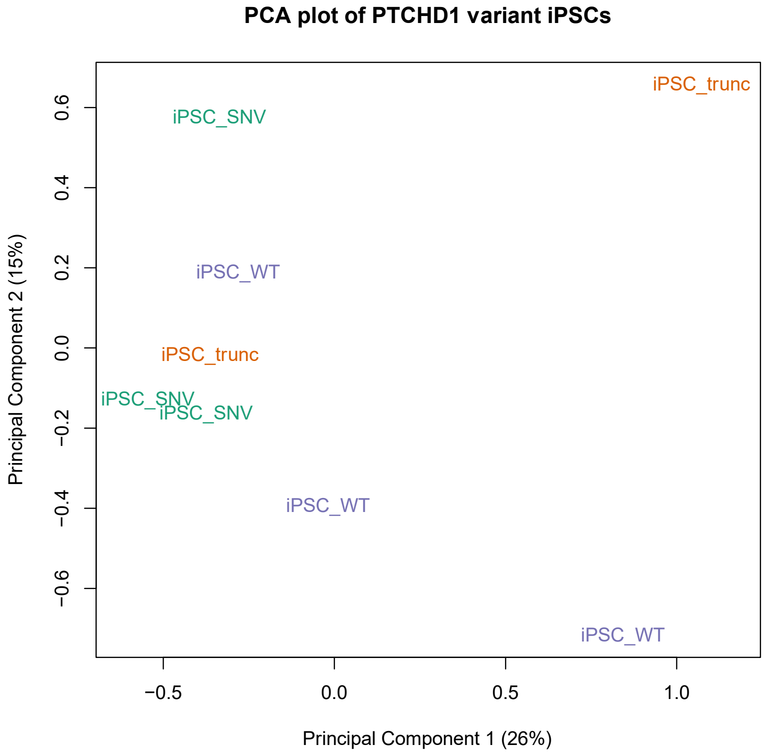
PCA plot of PTCHD1 variant iPSCs
iPSC_trunc
iPSC_SNV
iPSC_WT
iPSC_trunc
iPSC_SNV
iPSC_SNV
iPSC_WT
iPSC_WT
0.6
0.4
0.2
0.0
−0.2
−0.4
−0.6
−0.5
0.0
0.5
1.0
Principal Component 2 (15%)
Principal Component 1 (26%)

c.

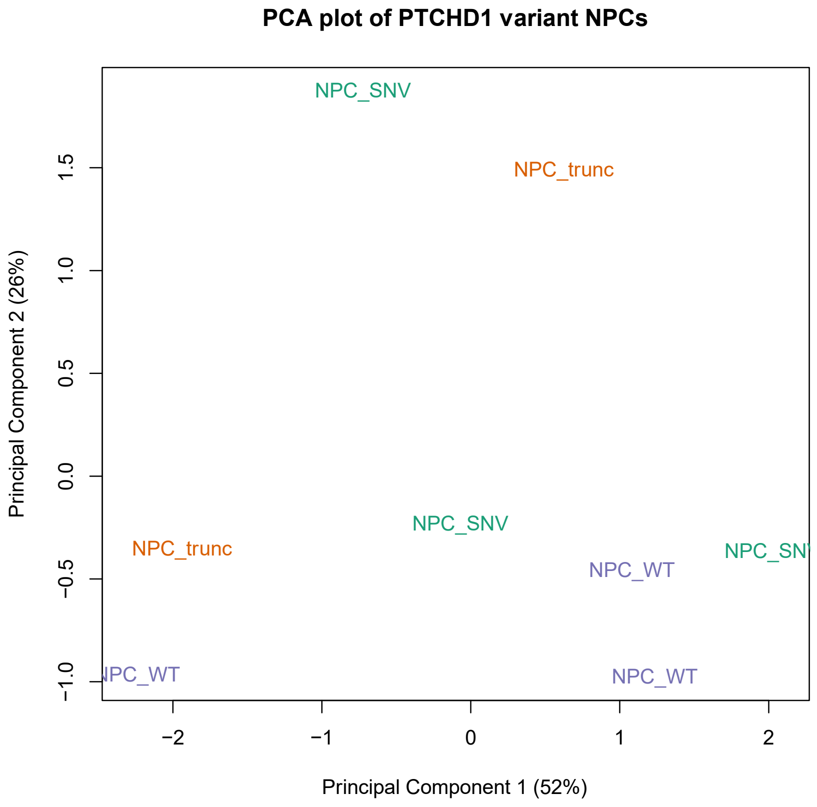

PCA plots show distances between gene expression profiles **a.** *PTCHD1*_WT, *PTCHD1*_SNV, and *PTCHD1*_trunc showed clear separation of iPSCs and NPCs. **b.** iPSCs **c.** NPCs

Figure S10

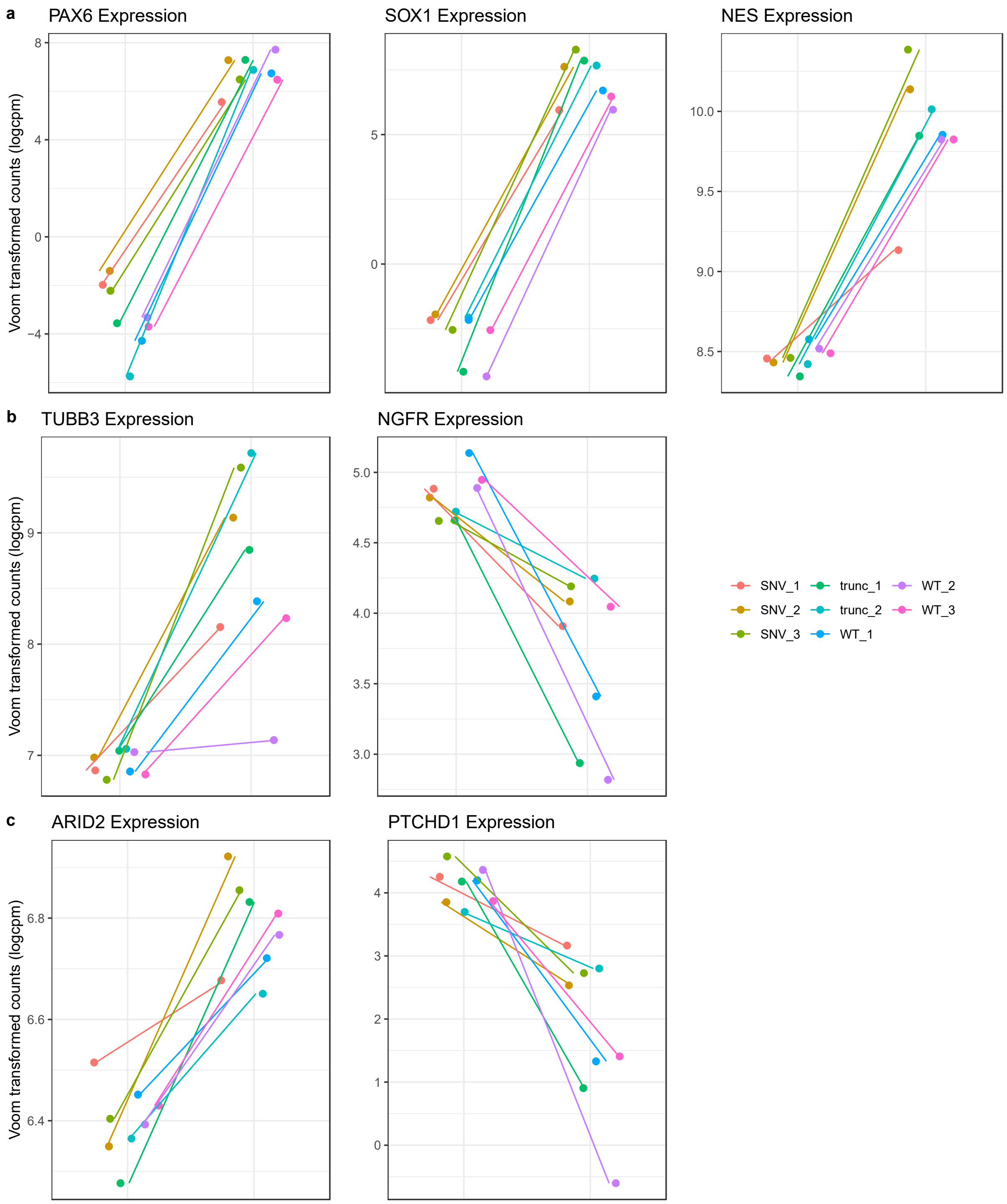

Marker/genes of interest expression

a NPC markers **b** mature neuron markers **c** genes of interest

Figure S11

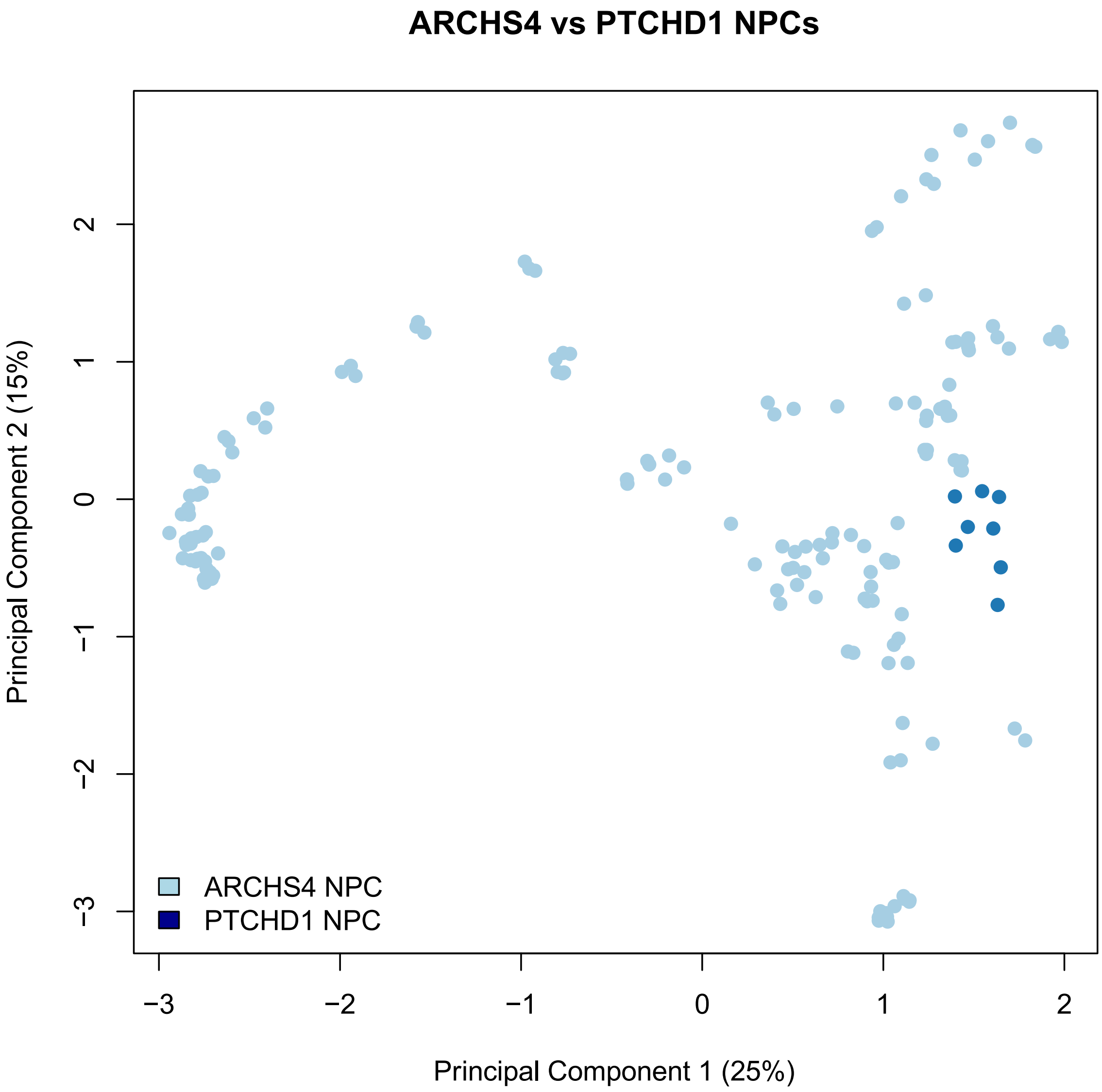

Principle component analysis of experimentally generated NPCs and wild-type NPCs in the ARCHS4 database.

Figure S12

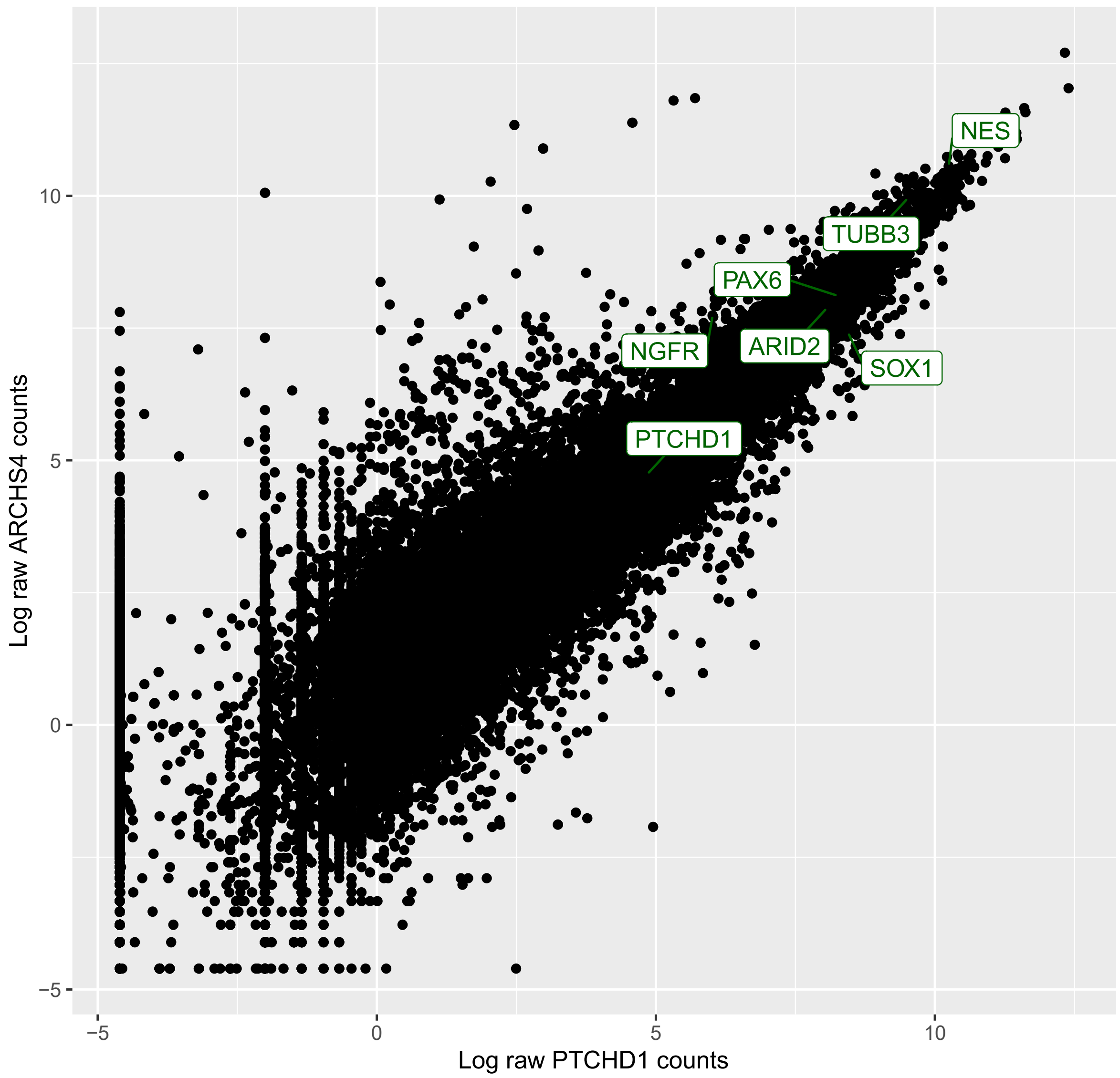

Dot plot of log-transformed mean raw counts for experimentally derived NPCs vs ARCHS4 wild-type NPCs.

Figure S13

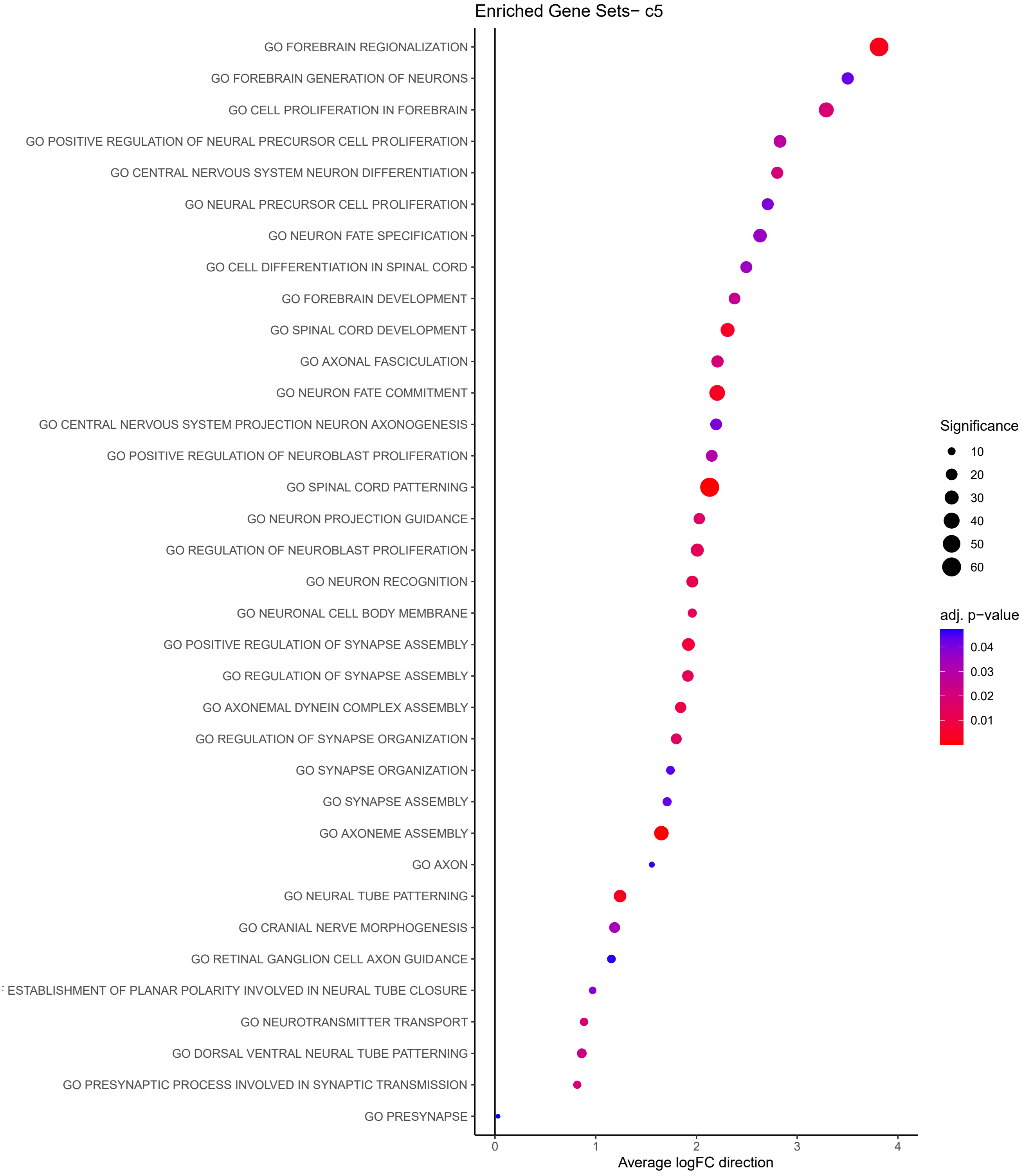

Gene sets commonly enriched during differentiation from the c5 Ontologies collection (GO and HPO)

Figure S14

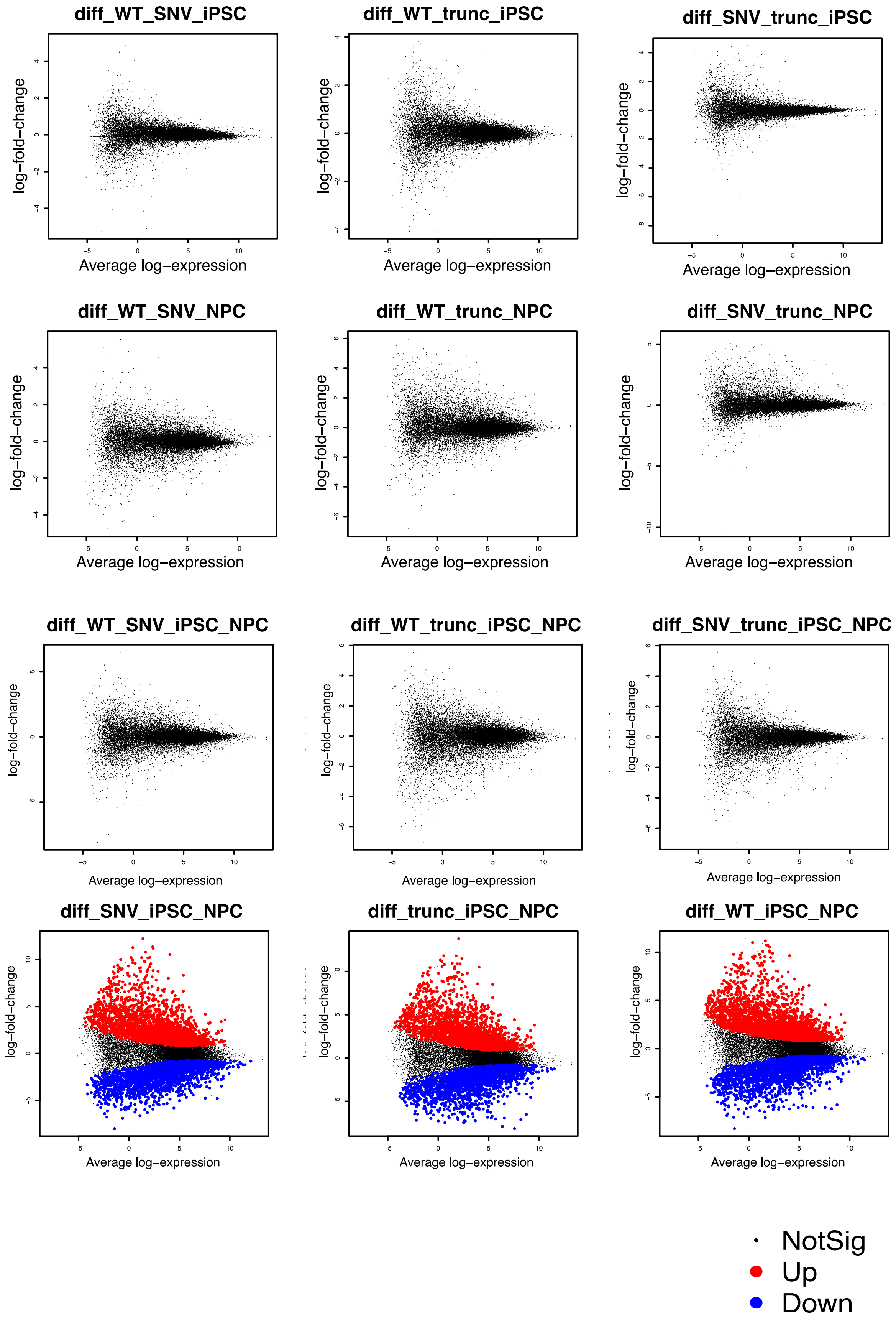

Mean-difference plots of *PTCHD1*_SNV, trunc and WT iPSCs, NPCs, and during differentiation

Figure S15

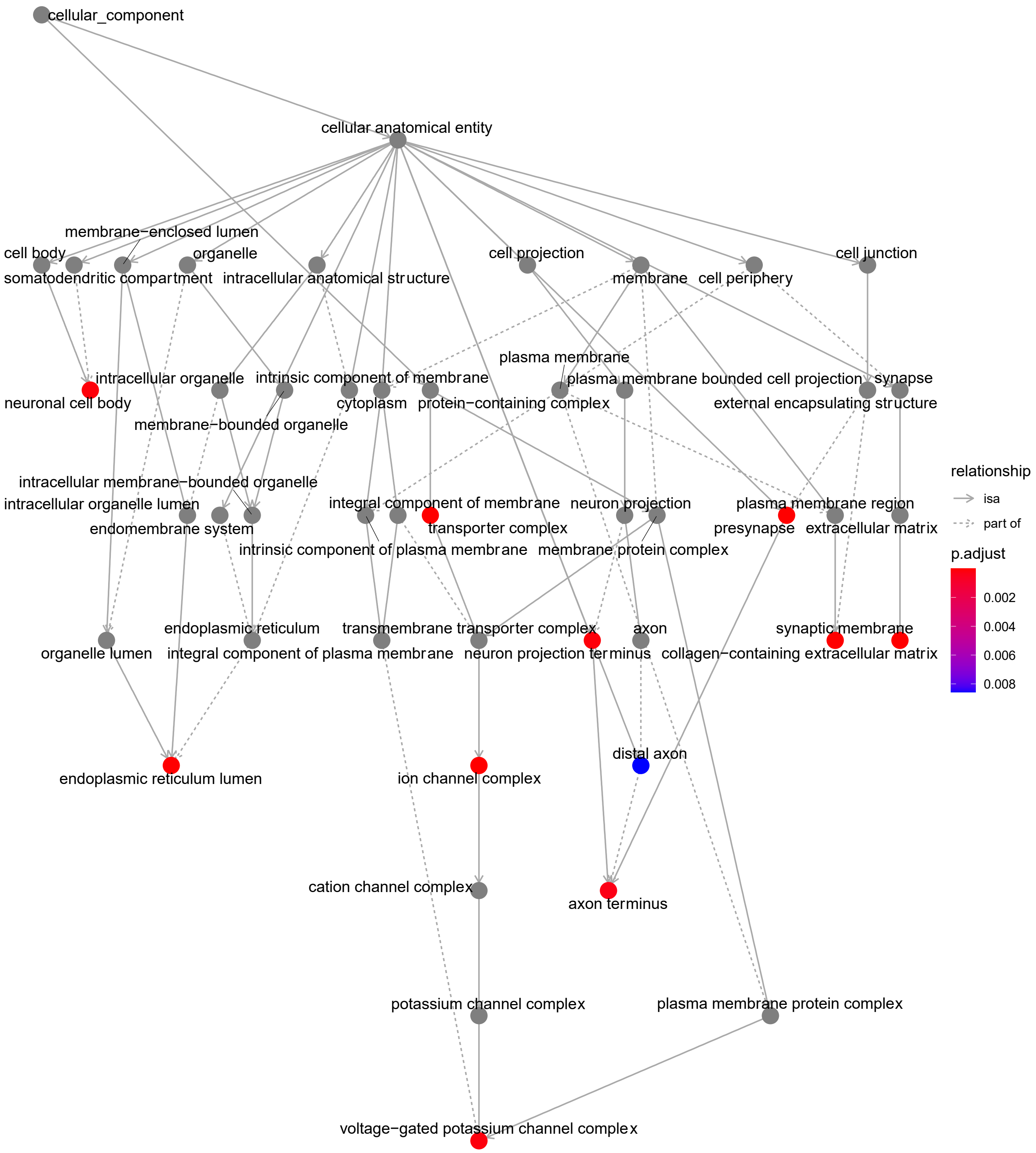

EnrichGO analysis identified synaptic cellular components were enriched in the comparison of *PTCHD1*_WT to *PTCHD1*_SNV cells during differentiation.

Figure S16

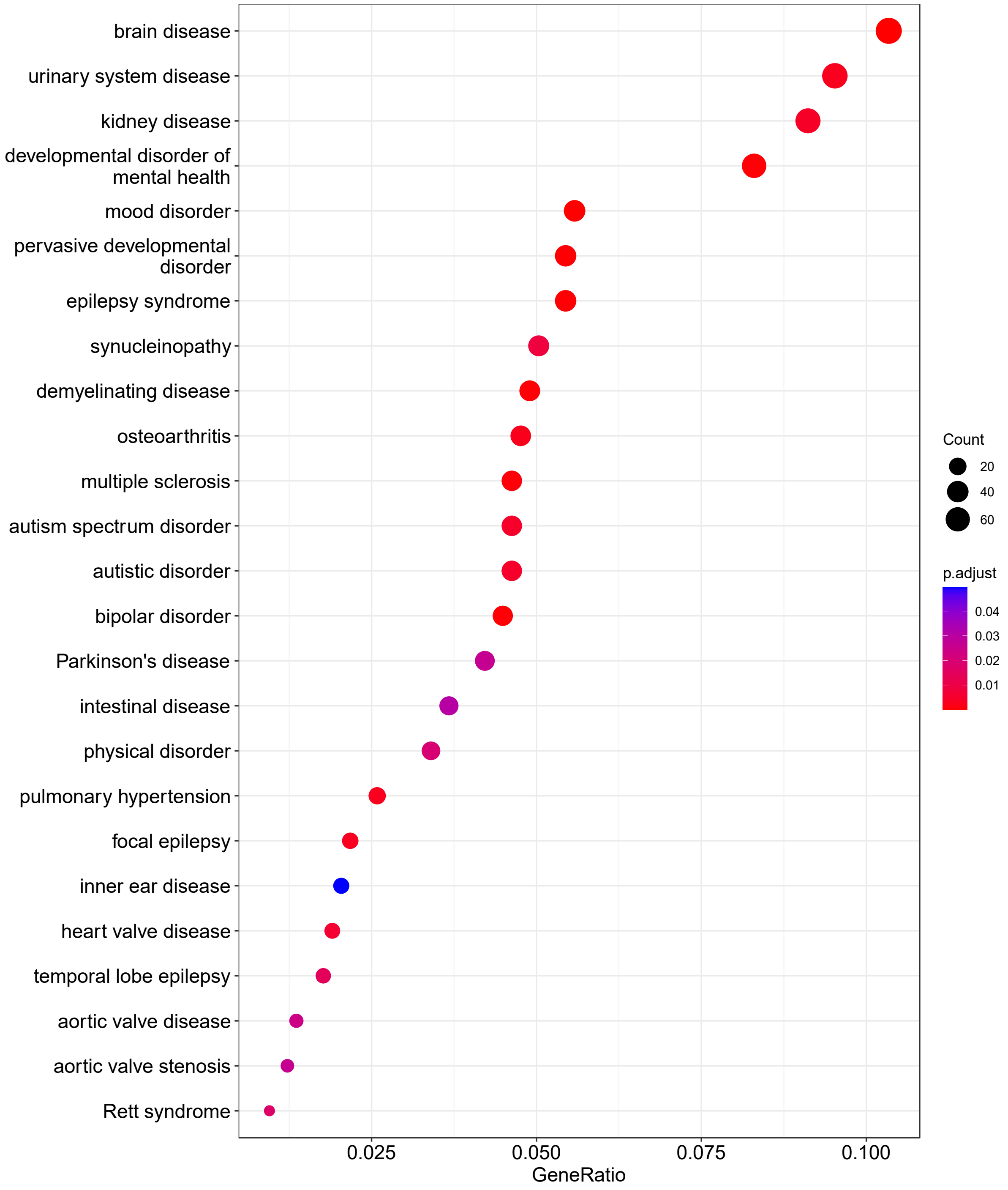

Enriched disease ontology terms from the comparison of *PTCHD1*_WT to *PTCHD1*_SNV cells during differentiation.

Figure S17

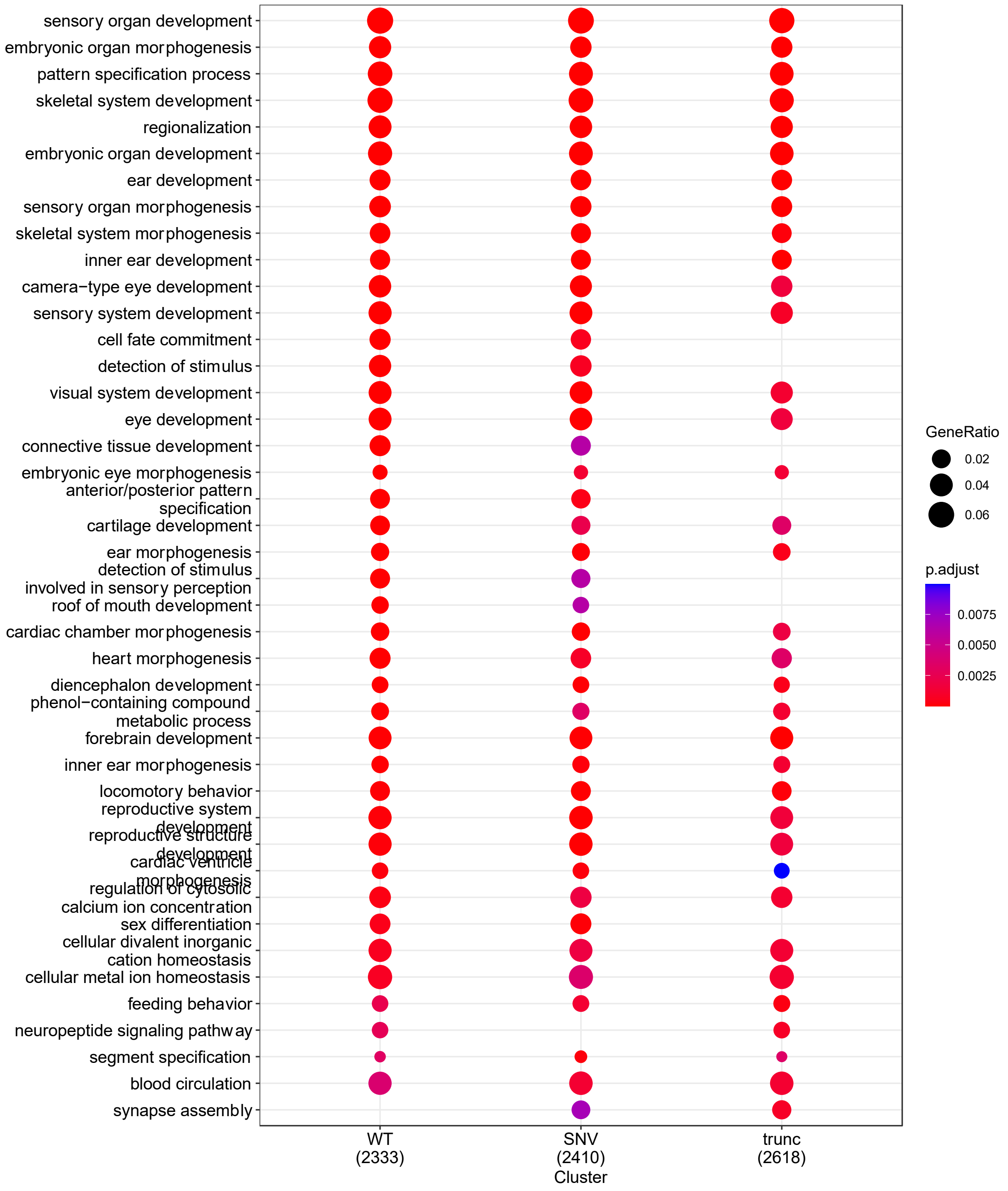

Clusterprofiler biological theme comparison of *PTCHD1*_WT, *PTCHD1*_SNV, and *PTCHD1*_trunc cells during differentiation.

Table S1

Human Phenotype Ontology (HPO) terms for patient with a VUS in the PTCHD1 gene (NM_173495 c.2489T>G (p.Ile830Arg)].).

**HPO term**	**Description**
HP:0000729	Autistic behavior
HP:0000343	Long philtrum
HP:0000278	Retrognathia
HP:0000463	Anteverted nares
HP:0000276	Long face
HP:0030799	Scaphocephaly
HP:0008070	Sparse hair
HP:0000954	Single transverse palmar crease
HP:0001263	Global developmental delay
HP:0012330	**Pyelonephritis NOT**
HP:0001250	**Seizures NOT**
HP:0011398	Central hypotonia
HP:0001317	**Abnormality of the cerebellum NOT**
HP:0000666	Horizontal Nystagmus
HP:0002194	Delayed gross motor development
HP:0001252	Muscular hypotonia

Table S3: Off-target sequences and primers and amplicon size for crRNAs used to introduce *PTCHD1*_SNV to iPSCs

**Target**	**Target sequence**	**PAM**	**#MM**	**Locus**	**F1**	**R1**	**Size**
**crRNA AA**	CAAGCACCTGAACAGTGTAC	AGG	N/A	chrX:-23393979			
PTCHD1_AA_OT1	C-AGGACTTGAACAGTGTAC	TAG	3	chr15:+33294863	AGCCCCATGATTCTGCCTAAC	CGTCCATGCACCATTCAAGC	499bp
PTCHD1_AA_OT2	CCATCACCTGAGCAGTGTAC	AGG	3	chrX:+20921939	TGTAAATGAACTGCAAGCATCT	GAATGAAAAACCAAACATCGTAT	300bp
PTCHD1_AA_OT3*	CATGCACCTAAACAGTGTAC	AGG	2	chr5:-105802190	ATCAAACCCCAGAAAGATGC	TCTGAGGTTGTATGGTTTTGGA	320bp
PTCHD1_AA_OT4	CCATCACCCGAACAATGTAC	AAG	4	chr2:-126352907	TTTTAAATGGTTCCTGTGCAT	GGTTGGATATTTGTCTCCTTGG	277bp
PTCHD1_AA_OT5	CAATAACCCTAACAGTGTAC	AAG	4	chr5:+23920392	TCCTCACTTACAAATGGGAGCT	GCCATAGTTTGCCAATCCCT	489bp
PTCHD1_AA_OT6*	ATAGCACATGAACAGTATAC	AGG	4	chr8:-3958051	TGATCTACTTCTCAGGGCAGC	TTTCTGCCTCCTTTTTGGTTTCC	491bp
**crRNA_Ax**	ATCTGACCTGTACACTGTTC	AGG	N/A	chrX:+23393970			
crRNA_Ax_OT1	ATCTGGGACGTGTACACTGTTC	CAG	3	chr3:+114957866	TTCATCGTGACAACCACACA	TCCCTGAAAAGGGAGCTTGAC	488bp
crRNA_Ax_OT2	ACCTGAACTTAACACTGTTC	AGG	4	chr11:-45192673	ATGAACCAGTTCTGGCTGGAG	CAGTGACACATCATCAACTATCCC	421bp
crRNA_Ax_OT3*	GTCTCAACTGTGCACTGTTC	CAG	4	chr13:-112440782	AAGATTGAGAGCCACACCCC	TGGTGTCCTGACACGTATTC	403bp
crRNA_Ax_OT4	ATTTCA-CTGTACACTGTTC	TAG	3	chr2:+158899895	CCATGTAAGGCCTGCATCTT	TGGGCACAGTTCTACCATGT	308bp
crRNA_Ax_OT5*	ATCTTAACTCTACACTGTTC	TAG	3	chr8:-98902909	ATCACCCTGAGCCCTCCATA	GATGGTGCTAAAGAGGCGGT	412bp
crRNA_Ax_OT6	CTCTAACCCTTACACTGTTC	AAG	4	chr11:-108314619	ACCCAGCCCATGTACAGTTT	GCCTAAAGTACATATCAACCAGC	559bp

Table S14: PTCHD1 patient variant of uncertain significance and in silico predictions of pathogenicity

Mutation	SIFT [1]	Polyphen 2 (Humdiv) [2]	FATHMM [3]	Residue Conservation Score [4,5]
**PTCHD1 I830R**	Not tolerated	Possibly damaging	Damaging	66

[1] https://sift.bii.a-star.edu.sg/

[2] http://genetics.bwh.harvard.edu/pph2/

[3] https://fathmm.biocompute.org.uk/

[4] https://pubmed.ncbi.nlm.nih.gov/11093265/

[5] https://pubmed.ncbi.nlm.nih.gov/12112692/
